# Combined analysis of mRNA and miRNA identifies dehydration and salinity responsive key molecular players in citrus roots

**DOI:** 10.1038/srep42094

**Published:** 2017-02-06

**Authors:** Rangjin Xie, Jin Zhang, Yanyan Ma, Xiaoting Pan, Cuicui Dong, Shaoping Pang, Shaolan He, Lie Deng, Shilai Yi, Yongqiang Zheng, Qiang Lv

**Affiliations:** 1 Citrus Research Institute, Southwest University/Chinese Academy of Agricultural Sciences, Chongqing, 400716, China

## Abstract

Citrus is one of the most economically important fruit crops around world. Drought and salinity stresses adversely affected its productivity and fruit quality. However, the genetic regulatory networks and signaling pathways involved in drought and salinity remain to be elucidated. With RNA-seq and sRNA-seq, an integrative analysis of miRNA and mRNA expression profiling and their regulatory networks were conducted using citrus roots subjected to dehydration and salt treatment. Differentially expressed (DE) mRNA and miRNA profiles were obtained according to fold change analysis and the relationships between miRNAs and target mRNAs were found to be coherent and incoherent in the regulatory networks. GO enrichment analysis revealed that some crucial biological processes related to signal transduction (e.g. ‘MAPK cascade’), hormone-mediated signaling pathways (e.g. abscisic acid- activated signaling pathway’), reactive oxygen species (ROS) metabolic process (e.g. ‘hydrogen peroxide catabolic process’) and transcription factors (e.g., ‘MYB, ZFP and bZIP’) were involved in dehydration and/or salt treatment. The molecular players in response to dehydration and salt treatment were partially overlapping. Quantitative reverse transcriptase-polymerase chain reaction (qRT-PCR) analysis further confirmed the results from RNA-seq and sRNA-seq analysis. This study provides new insights into the molecular mechanisms how citrus roots respond to dehydration and salt treatment.

Around the world, drought and salinity as two major concerns for agriculture negatively affect plant growth and development, which ultimately lead to a decline in yield and quality^1^. Due to high salinity and drought, a great amount of land is unsuitable for plant growth. Fortunately, plants have evolved a series of sophisticated mechanisms to deal with these unfavorable conditions at cellular, physiological, molecular and biochemical levels^2^,^3^. In recent decades, a large number of efforts have been performed to elucidate the molecular mechanisms underlying plant adaptation to drought and salinity stress, and it has been well established that gene expression regulation at transcriptional and post-transcriptional is an important strategy for plants to combat these two stresses^4^. However, the molecular events how to regulate gene expression are far from clear.

MicroRNAs (miRNAs), as important molecular players for gene expression regulation, have attracted so much attention during recent years. It has been well known that miRNAs are a type of small non-coding RNAs with 21–24 nt in length and negatively modulate the expression of their target genes by mRNA cleavage or translation repression^5^,^6^. According to the newest miRNA database (http://www.mirbase.org), a total of 35828 mature miRNA, to date, have been identified from 223 species, of which 8496 were included in 73 plant species. A large body of experimental data have indicated that miRNAs play crucial roles in diverse biological processes, including organ development^7^,^8^,^9^, cell proliferation^9^,^10^, developmental timing^11^, hormone signaling^12^ and stress response^4^,^13^,^14^. Of them, the roles in response to stresses are one aspect of currently active research. Early studies show that miRNAs are implicated in a wide variety of stresses including heat^15^, drought, salinity^4^, heavy metal^16^, chilling temperature^17^, nutrient stress^18^ and disease^19^. In plants, more than 40 miRNA families have been reported to play critical roles in abiotic stresses, many of them involved in salt and drought stress response^4^. Some miRNAs, such as miRNA156, miRNA169, miRNA173, miRNA394, miRNA395 and miRNA396, have been identified in a series of plant species, indicating that their function in the response to stresses might be conserved among plants^4^,^20^.

Citrus is the most economically important fruit crop in the world. However, the productivity and fruit quality are adversely affected by drought and salinity stress^21^. Thus, improvement of tolerance to these two stresses can reduce economic loss to citrus growers. Experimental data show that drought and salinity can negatively affect citrus numerous biological and metabolic pathways, including photosynthesis, carbon fixation, ROS as well as respiration^22^,^23^, just as reflected at molecular level that a very large number of genes have been involved. The similar cases were observed in other plant species, such as maize^24^, cotton^4^, Arabidopsis^25^, as well as switchgrass^26^. For instance, over-expression of a citrus *CrNCED1* gene in transgenetic tobacco resulted in improved tolerance to drought, salt and oxidative stresses, showing CrNCED1 might be an important regulator to fight drought and salt stress in citrus^27^. Similarly, transgenic tobacco over-expressing the sweet orange glutathione transferase (CsGSTU) genes (*CsGSTU1* and *CsGSTU2*) exhibited stronger tolerance to drought and salt stress^28^. Recently, by genome-wide analysis, some salt- and drought- signal transduction pathways in citrus have been discovered, in which numerous candidate genes are expressed differentially, and have great potential to enhance tolerance to salt and drought stress, such as R2R3MYB, NAC and polyamine oxidase^29^,^30^,^31^. Although a great number of progresses have been made in citrus, the mechanisms controlling citrus response to salt and drought stress remain unclear.

As a critical regulatory player, miRNAs have an important role during citrus growth and development or under stresses. In recent years, using computational and sequencing technology, numerous conserved and new miRNAs have been identified in citrus^32^,^33^,^34^,^35^,^36^,^37^,^38^. These data have unraveled that miRNAs are involved in nutrient deficiency^36^,^37^, pathogen infection^35^, mal sterility^34^, and somatic embryogenesis^38^. However, no information, to date, is available about how miRNAs are involved in salt and drought stress. In this study, we used RNA-seq and miRNA-seq to identify miRNAs and mRNAs that differentially expressed under salt and dehydration treatment. As expected, we have identified a large number of genes, transcription factors and miRNAs to be involved in the regulation of salt and dehydration response. The results of this study provided a deep insight into the molecular mechanisms how citrus roots fight salt and dehydration stress, which will contribute to improve tolerance of citrus to these two stresses in future.

## Results

### mRNA sequencing data mapping and annotation

**Figure 1:**
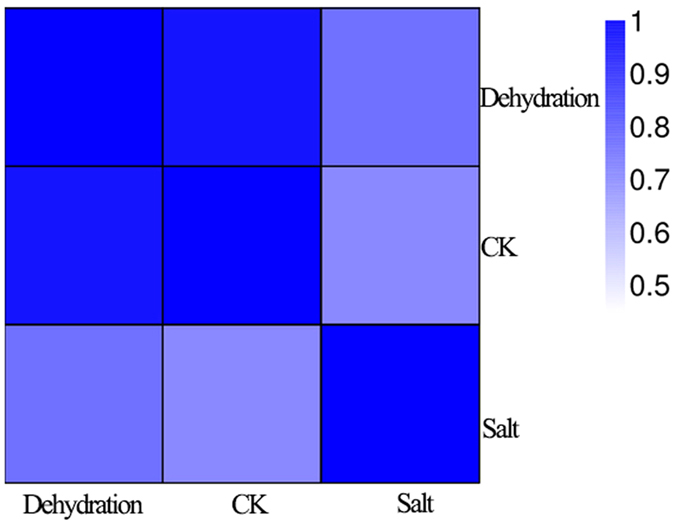
The correlation between each two samples based on FPKM result.

A total of 3 cDNA libraries from the control (0 h), dehydration- (1 h) and salt- (24 h) treated roots, referred as to CK, DR and SA, respectively, were sequenced. Overviews of the sequencing and assembly results were listed in Table 1. After removing the low-quality raw reads, RNA-seq produced 42,468,660, 34,424,826 and 37,931,432 clean reads for CK, DR and SA sample, accounting for more than 99.12%, 99.06% and 99.17%, respectively. After mapping clean reads to the clementina genome, approximately 83.26% (DR)–83.91% (SA) reads were successfully aligned, with 72.87–73.81% of reads mapped to CDS regions, and 3.19–3.73% of reads mapped to introns or intergenic regions, while 1.87–2.12% of reads had multiple alignments. The correlation value between SA and DR was over more than 0.75 (Fig. 1), indicating the molecular players in response to dehydrate and salt were partially overlapping.

### miRNA sequencing data mapping and annotation

**Figure 2:**
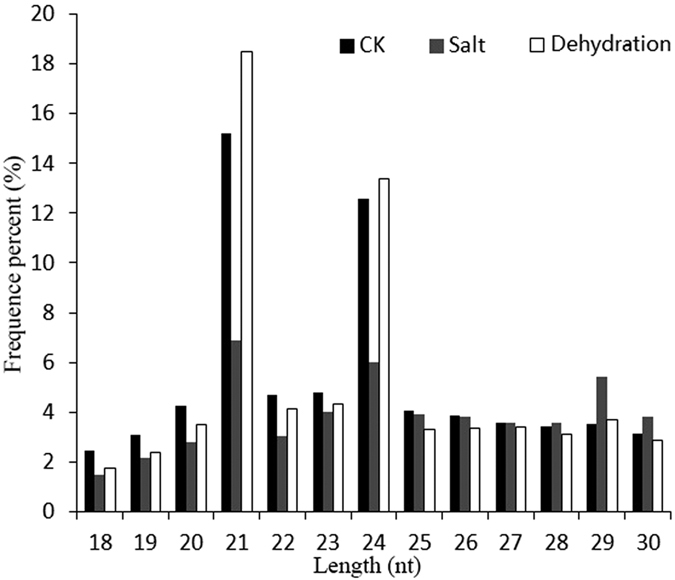
Length (nt) distribution of sRNAs.

Three small RNA libraries were constructed using citrus roots with or without dehydration and salt treatment (Table 2). A total of 18,140,473 raw reads were obtained from the CK sample, 22,152,310 raw reads from DR sample and 25,460,679 raw reads from SA sample. After removing reads with non-canonical letters or with low quality, the 3’ adapter was trimmed and the sequences shorter than 18 nt were also discarded. In finally, 16,552,632, 19,881,239 and 23,441,245 million clean reads were yielded in CK, DR and SA sample, respectively, and most of them were between 21–24 nt in length, and the read counts with 21 nt were highest (Fig. 2), followed by 24 nt, which was in line with previous reports on *Arabidopsis*^39^, grapevine^40^, tea^41^ and rice^42^. A total of 391 mature miRNAs were identified. Of them, 149 were annotated citrus miRNAs already present in miRbase v20.0, while 242 were novel miRNAs not homologous to any other species (Table S3 and Figure S1).

### DE genes in response to dehydration and salt treatment

In this study, RNA-seq yielded 21700, 21595 and 21202 genes in CK, DR and SA sample, respectively. With a criteria of at least a 2 fold difference and a p-value less than 0.05 (|log2FC| ≥ 1, p < 0.05), a total of 1396 and 1644 genes were differentially expressed in response to dehydration and salt, respectively. Of them, 466 DE genes were overlapped, more than 91.6% of which with similar expression patterns, indicating the molecular basis of dehydration tolerance was, at least in part, common to that of salt tolerance. Of the 2574 DE genes, 1951 genes were well annotated on the clementina genome (Cclementina_182_v1.0), of which 692 genes being up-regulated and 952 genes down-regulated in the SA sample, and 1022 genes up-regulated and 374 genes down-regulated in DR sample.

**Figure 3:**
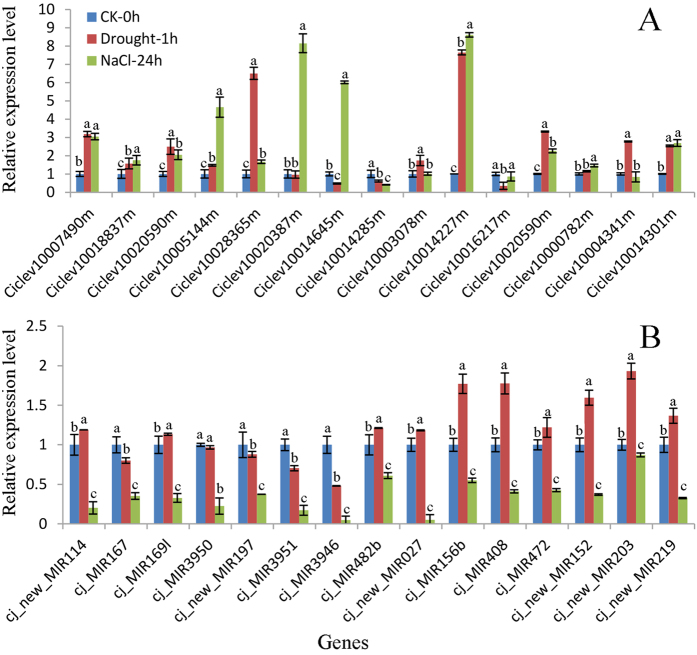
Results from qRT-PCR of miRNAs and mRNAs in *Citrus junos*. sRNAs and mRNAs were isolated from roots treated with dehydration and salt, respectively. The expression levels of miRNAs and mRNAs were normalized to U6 snRNA and Actin gene, respectively. The mormalized miRNA and mRNA levels in the control were arbitrarily set to 1.

To validate the RNA-seq results, 15 genes were selected for qRT-PCR analysis (Fig. 3A). Compared with the control, the expression of *S-locus lectin protein kinase* (Ciclev10007490m), *Leucine-rich repeat protein kinase* (Ciclev10018837m), *Calcineurin-like phosphoesterase* (Ciclev10020590), *nuclear factor Y, subunit A1* (Ciclev10005144m), *Transducin/WD40 repeat-like* (Ciclev10028365m), *P-loop containing nucleoside triphosphate hydrolase* (Ciclev10020387m), *amino acid transporter 1* (Ciclev10014645m) and the gene with unknown function (Ciclev10003078m), *ATPase E1-E2* (Ciclev10014301m), *sucrose synthase* (Ciclev10004341m), *thiamin biosynthesis protein* (Ciclev10000782m), *Calcineurin-like phosphoesterase* (Ciclev10020590m), *unknown function protein* (Ciclev10016217m), *glutamate receptor* (Ciclev10014227m) were all up-regulated by salt or dehydration or both. As expected, the expression of *glutamate receptor* (Ciclev10014285m) was down-regulated by salt and dehydration treatment. Based on the above results, the qRT-PCR analyses, in large part, confirmed the reliability RNA-seq data, indicating the reliability of the RNA-seq analysis.

### DE miRNAs in response to dehydration and salt treatment

In the miRNA-seq data, a total of 76 DE miRNAs were identified in SA and DR sample with a criteria of at least a 1.5 fold difference and total reads count no less than 20 (|logFC| ≥ 1, total ≥ 20, p ≤ 0.05), of which 29 belonged to novel miRNAs (Table 3). There were 19 known miRNAs and 15 novel miRNAs in response to dehydration treatment, of which 16 were down-regulated and 18 were up-regulated. Forty-one known miRANs and 21 novel miRNAs differentially expressed in the SA samples, of them, 58 miRNAs were down-regulated and 4 were up-regulated. Of 76 DE miRNAs, 21 of them were overlapped in salt and dehydration samples, i.e. cj_MIR164, cj_MIR390, cj_MIR393b, cj_MIR3950, cj_MIR3951, cj_MIR396, cj_MIR397, cj_MIR398, cj_MIR398b, cj_MIR399d, cj_MIR408, cj_MIR482b, cj_MIR482c, cj_MIR535, cj_new_MIR027, cj_new_MIR055, cj_new_MIR065, cj_new_MIR108, cj_new_MIR145, cj_new_MIR152 and cj_new_MIR197. As expected, these overlapping DE miRNAs with the exception of cj_MIR390, cj_MIR393b and cj_MIR482b exhibited similar expression patterns under SA and DR treatments, further demonstrating the common molecular basis underlying dehydration and salt tolerance.

To validate the miRNA sequencing, 15 miRNAs i.e. cj_MIR156b, cj_MIR167, cj_MIR169l, cj_MIR3946, cj_MIR3950, cj_MIR3951, cj_MIR408, cj_MIR472, cj_MIR482b, cj_new_MIR152, cj_new_MIR203, cj_new_MIR219, cj_new_MIR197, cj_new_MIR027 and cj_new_MIR114 were selected for qRT-PCR analysis (Fig. 3B). Compared to the control, expression of cj_MIR3946, cj_MIR3951 and cj_new_MIR197 were all down-regulated by salt and dehydration treatment, whereas expression of cj_MIR156b, cj_MIR408, cj_MIR472, cj_new_MIR152, cj_new_MIR203, cj_new_MIR219 and cj_MIR482b was up-regulated by drought and down-regulated by salt treatment. These data with the exception of cj_new_MIR027 were in line with the results of miRNA-seq, showing the reliability of miRNA-seq analysis.

### Pathway analysis of DE genes

**Figure 4:**
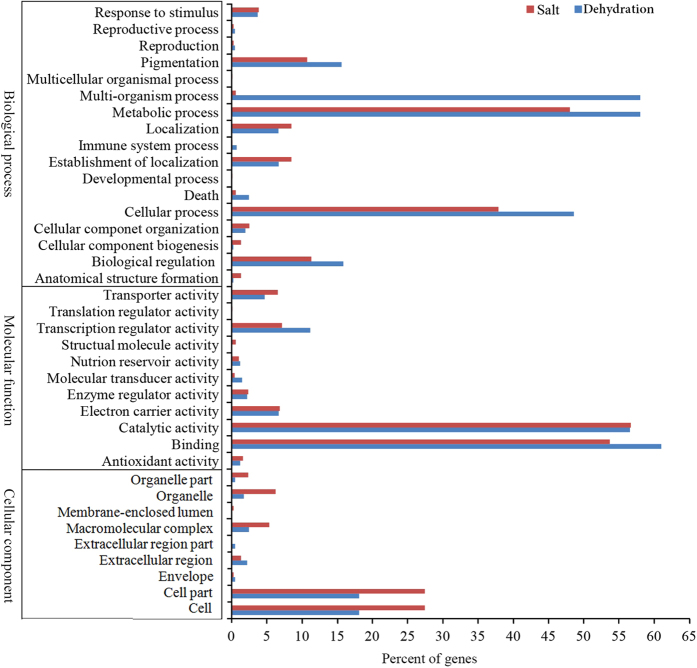
Functional categorization of significantly differentially expressed genes in *Citrus junos* roots under dehydration (blue column) and salt stress (red column). Functional categorization was performed with BGI WEGO.

The functional classification of DE mRNAs was performed with GO term and KEGG pathway enrichment analysis with aim to elucidate the biological processes/pathways and the relationship between salt- and dehydration-response. GO enrichment analysis revealed that some crucial biological processes related to carbohydrate metabolic processes (e.g. ‘glucan and polyanime biosynthetic process’) (Table 4), reactive oxygen species (ROS) metabolic process (e.g. ‘hydrogen peroxide catabolic process’) (Table 5) and transcription factors (e.g., ‘MYB, ZFP and bZIP’) (Table 6) were distinct between SA and DR samples, while several important GO terms, for example signal transduction (e.g. ‘MAPK cascade’) and hormone-mediated signaling pathways (e.g. abscisic acid- activated signaling pathway’) were overlapped in both treatment samples (Fig. 4). In this study, a total of 94 pathways that changed significantly (p ≤ 0.05) after salt- and dehydration- treatment were identified by KEGG pathway analysis. Of them, 50 pathways overlapped including ‘Plant hormone signal transduction’, ‘Starch and sucrose metabolism’, ‘Phenylalanine, tyrosine and tryptophan biosynthesis’ and ‘Arginine and proline metabolism’, and 37 pathways (e.g. ‘Citrate cycle’, ‘Nirogen metabolism’, and ‘Ascorbate and aldarate metabolism’) were specific to salt treatment and 7 specific to drought treatment, including ‘Valine, leucine and isoleucine biosynthesis’, ‘Zeatin biosynthesis’ and ‘Glycosphingolipid biosynthesis’. These results indicated that the DE genes obtained in this study might play crucial roles in salt- and dehydration-stress in citrus plants.

### Pathway analysis of DE miRNAs

**Figure 5:**
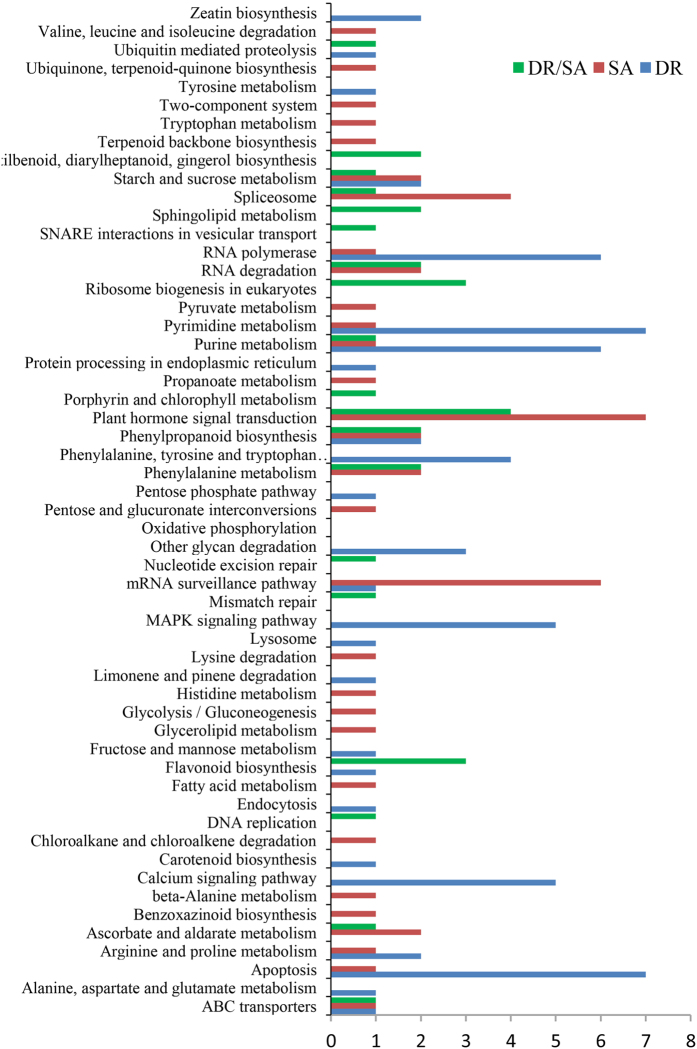
Pathway enrichment analysis of significantly differentially expressed genes in *Citrus junos* roots under dehydration and salt stress.

By miRNA-targeted pathway union analysis, there were 55 KEGG pathways significantly (Fisher Exact Probability Test, p < 0.05) related with genes targeted by DE miRNAs (Fig. 5). Numerous pathways including the plant hormone signal transduction, oxidative phosphorylation, ascorbate and aldarate metabolism, flavonoid biosynthesis and phenylalanine metabolism were involved in salt and dehydration response. It was worthy to note that some pathways were especially involved in dehydration stress including calcium signaling pathway, MAPK signaling pathway and zeatin biosynthesis, and some pathways such as tryptophan metabolism, propanoate metabolism and fatty acid metabolism were only responded to salt treatment.

### Correlation of DE miRNAs and mRNAs in response to dehydration and salt stress

**Figure 6:**
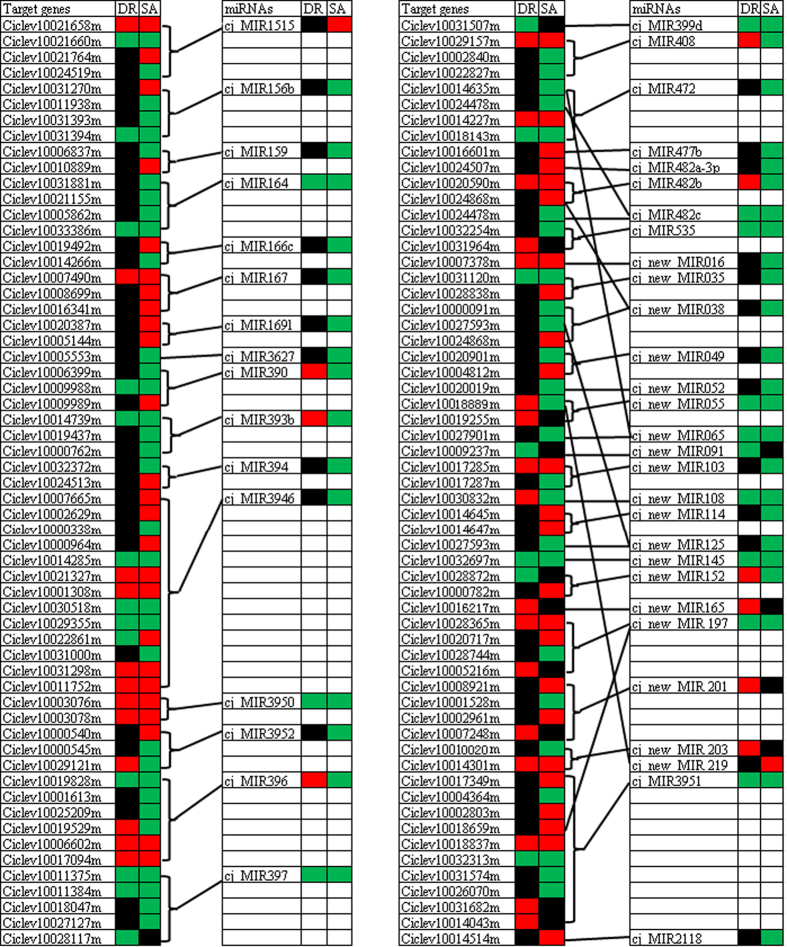
miRNA-mRNA correlation network. DR and SA indicate dehydration and salt treatment, respectively. Down-regulated mRNAs and miRNAs were shown as green and the up-regulated shown as red.

The miRNA-gene interactions between DE miRNAs and DE mRNAs were investigated with an in-house R script. The results showed that 121 miRNA-mRNA interactions significantly responded to draught and salt treatment were identified, of which 21 DE miRNAs and 48 DE mRNAs were involved in dehydration treatment, and 41 DE miRNAs and 108 DE mRNAs were implicated in salt treatment (Table S1 and Fig. 6). Additionally, there were 3 DE miRNAs responding to dehydration and salt treatment, while their target mRNAs were just responded to one stimulus. For instance, although cj_MIR399d was down-regulated by dehydration and salt treatments, its target gene i.e. Ciclev10031507m was just down-regulated by dehydration. Since miRNAs negatively regulate the expression of their target genes by target mRNA cleavage, the expression patterns of miRNAs generally show an opposite trend to those of their target genes. According to this theory, the DE miRNA that involve target gene cleavage were induced by salt or/and drought treatment, their target mRNAs are reduced, vice versa. As expected, 9 significantly down-regulated miRNAs, in this study, showed inverse expression pattern to their DE target genes. However, some DE miRNA such as cj_MIR1515, cj_MIR156b and cj_MIR159 showed positive and negative relationships with its target genes. From Fig. 6, our data showed that a single miRNA such as cj_MIR394, cj_MIR3946 and cj_MIR3951 can regulate multiple target mRNAs and vice versa. These results indicated the miRNA-mRNA regulatory network involved in dehydration and salt treatment was more complex than previously thought. GO annotation of 14 deregulated target mRNAs in response to draught and salt treatments revealed that the important roles in ‘tryptophan biosynthesis’, ‘perception of the hormone’, ‘regulation of transcription, and ‘plant immunity’ (Table S1).

### Experimental validation of miRNA-guided cleavage of target mRNA

**Figure 7:**
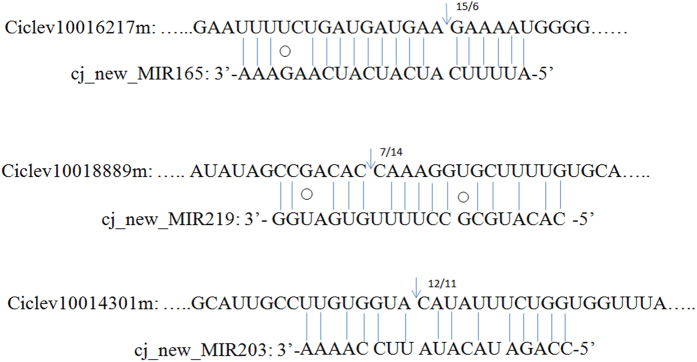
Mapping of the mRNA cleavage sites by RNA ligase-mediated 5′ RANC. Watson-Crick pairing was indicated by vertical dashes and G:U wobble paring by circles. The arrows indicated the 5′ termini of mRNA fragments isolated from roots of *Citrus junos* Siebold cv. ‘Ziyang’, as identified by cloned 5′RACE products, with the frequency of clones shown. RNA ligase-mediated 5′RACE was used to map the cleavage sites. The partial mRNA sequences from the target genes were aligned with the miRNAs.

It is widely accepted that miRNA-mediated gene silencing in plants is the direct cleavage of target mRNA through binding to coding sequence with near-perfect complementarity^43^. The RNA ligase-mediated 5′ RACE (RLM-5′ RACE) can readily detect this cleavage, which have validated many predicted miRNA targets for most of Arabidopsis miRNA families^44^. In order to testify whether DE miRNAs can mediate the cleavage of their predicted targets, RLM-5′ RACE was conducted on predicted targets for, respectively. The results revealed that the *Ciclev10016217, Ciclev10014301* and *Ciclev10018889* are indeed cleaved by the potential cj_new_MIR165, cj_new_MIR203 and cj_new_MIR219, respectively (Figure Fig. 7). Further study should be performed to identify target cleavage sites, which can be helpful in understanding small RNA-mediated gene regulation in citrus plants.

## Discussion

In this study, our work firstly provided a detailed snapshot of parallel mRNA and miRNA expression levels in citrus plants under dehydration and salt treatment, which helped us dissect the molecular mechanisms underlying drought and salinity tolerance. By integrative analysis, we obtained a set of dehydration- and salt-responsive mRNAs/miRNAs, mRNA-miRNA interactions and the differences in biological processes/pathways between dehydration and salt treatment, which helped us understand the differences between dehydration and salinity response mechanisms and simultaneously provide numerous potential genes to enhance drought and salinity tolerance of citrus plants in future.

Several previous studies have demonstrated that the stress-responsive miRNA-mRNA regulatory networks exhibited coherent and incoherent regulatory patterns^41^,^45^. Likewise, in this study, we successfully constructed 121 miRNA-mRNA pairs, of which both negative and positive correlations were also found (Table S1 and Fig. 6). In general, the negative correlation between miRNA and its target mRNA is a considered proof of miRNA targeting, but a few cases with positive correlation have also been reported^41^,^46^. More recently, several reports have demonstrated that miRNA targets have a negative or positive feedback regulation on their respective miRNAs^47^,^48^, which could also provide an explanation to the incoherent correlations between miRNA and its targets in this study. In addition, our data showed that a single miRNA could target multiple mRNA, and vice versa, exhibiting a more complex miRNA-mRNA regulatory network than we had believed before. Zheng *et al*.^41^ suggest that these miRNAs are response for both switch on/off and fine-tune target mRNA expression under stresses.

Based on GO and KEGG analysis, the functional and pathway assignments of DE mRNAs and DE miRNAs-mediated targets showed that a number of metabolic, physiological, and hormonal responses were involved in dehydration and salt stresses in citrus roots, which included carbohydrate metabolism, plant hormone signal transduction, protein phosphorylation and transcription factors (Fig. 4 and Table 6).

Under abiotic stresses such as drought, cold and salinity, the soluble carbohydrates will rapidly be accumulated in plants. Starch as the main carbohydrate store in most plants can be rapidly mobilized to provide soluble sugars which are very sensitive to changes in the environment. ß-amylase (BMY) is a key enzyme involved to starch degradation^1^. Osmotic stress could increase total b-amylase activity and decrease light-stimulated starch content in wild-type Arabidopsis but not in *bam1 (bmy7*) mutants, which appeared to be hypersensitive to osmotic stress^49^. Similarly, 3 *BYM* members, here, were found to respond to dehydration, but not to salt treatment (Table 4), which was in line with previous reports. Besides starch, trehalose has a potential role in plant stress tolerance^50^, which is synthesized in a two-step linear pathway in which trehalose-6-phosphate synthase (TPS) generates trehalose-6-phosphate (T6P) from UDP-glucose and glucose-6-phosphate followed by dephosphorylation to trehalose by trehalose-6-phosphate phosphatase (TPP)^51^. Over-expression of different isoforms of *TPS* from rice conferred enhanced resistance to salinity, cold, and/or drought^52^. As expected, one *TPS* gene, here, was up-regulated by both dehydration and salt. Raffinose family oligosaccharides (RFO) including raffinose, stachyose, and verbascose significantly accumulate in leaves of plants experiencing environmental stress such as cold, drought or high salinity^53^,^54^,^55^,^56^. GolS (galactinol synthase) and StS (Stachyose synthase) are two important enzymes in RFO pathway. In this study, two *GolS* members and two *StS* members were positively responded to dehydration or/and salt. In Arabidopsis, over-expressing *GolS* lead to accumulating high levels of galactinol and raffinose and more tolerant to drought and salinity stress^54^,^56^. However, upon *StS* gene, no data, to date, is available, which remains to be elucidated.

Polyamines (PA) play important functions in the regulation of abiotic stress tolerance such as drought, salinity, wounding as well as temperature extremes^57^. There are several key enzymes involving in PA pathway including ornithine decarboxylase (ODC), arginine decarboxylase (ADC), spermidine synthase (SPDS), spermine synthase (SPMS) and polyamine-oxidases (PAOs). *Arabidopsis* plants deficient in ADC2 have reduced putrescine level and were hypersensitive to salt stress^58^, and up-regulation of ADC led to an increase in putrescine level and enhanced drought tolerance^59^,^60^, showing the important roles of *ADC* genes in drought and salt stress. In this study, an *ADC* gene (*ADC1*) was up-regulated by dehydration, whereas no one was responded to salt stress (Table 4). These results indicated that the functions of *ADC* genes from different plants were varied. In citrus, *ADC* genes were more important for drought tolerance than that of salt. Besides *ADC*, three *PAO* genes including *PAO1, PAO4* and *PAO5* were negatively or positively responded to salt or/and drought stresses. Briefly, the expression level of *PAO4* was increased under drought and salt stresses, while *PAO1* and *PAO5* just were up-regulated by salt stress (Table 4). Although a stimulation of polyamine oxidation was associated with the plant response to drought, salinity, osmotic stress and heat stress^61^, the roles of *PAOs* in response to drought and salt stresses remains elusive.

Since the GS/GOGAT pathway in plants was discovered in the 1970 s, the role of GDH in ammonium assimilation remains controversial. GDH may play a complementary role to the usual GS/GOGAT pathway in the re-assimilation of excess ammonia released under stress or intracellular hyper-ammonia conditions^62^. The GDH activity in salt-sensitive rice cultivars was lower than that of salt tolerance ones with increased salinity concentration^63^. Similar results were obtained in ammonium-tolerant pea (*Pisum sativum*) plants by Lasa *et al*.^64^. Recently, over-expression of a *GDH* gene from *Magnaporthe grisea* conferred dehydration tolerance to transgenic rice^62^. These results indicated that *GDH* genes may be involved in salt and drought stress. Our data, here, showed that there was one citrus *GDH* gene (Ciclev10031681m) just responded to salt stress, but not to drought (Table 4).

Under various environmental stresses, plants often generate reactive oxygen species (ROS) which generally lead to membrane lipid peroxidation and yield highly cytotoxic products of oxidative DNA damage^65^. Therefore, ROS homeostasis is of importance for plant to protect normal metabolism. Plants can fine-tune ROS levels through ROS scavenging enzymes, such as SOD, GST and POD^66^. As expected, our data showed that there were lots of ROS scavenging enzymes including glutathione S-transferases (GST), Peroxidases (POD) and Thioredoxins (Trx) were responded to salt and/or dehydration treatment (Table 5), which could have active functions to protect citrus roots from damage caused by salt and dehydration stress.

Abscisic acid (ABA) serves as an integral regulator of abiotic stress signaling, which can quickly accumulate under various environmental stresses^1^. In this study, several key genes involved in ABA biosynthesis and catabolism were remarkably up-regulated by drought and salt stress, suggesting its important roles in stresses tolerance (Table 5). In *Arabidopsis*, the *atabcg25* mutants are more sensitive to exogenous ABA, contrarily over-expressing *AtABCG25* led to ABA-insensitive transgenic plants^67^. Subsequently, biochemical analyses showed that AtABCG25 mediates ATP-dependent ABA efflux from the cytosol to the extracellular space^67^. In this study, an *ABC* gene (Ciclev10011273m), the *AtABCG25* homolog, was significantly down-regulated by salt and drought stress. This result indicated that the translocation of endogenous ABA from cytosol to extracellular space was inhibited when citrus roots were subjected to dehydration and salt stress, which thereby increased the tolerance to these two abiotic stresses. *PP2C* genes acting as negative or positive regulators of ABA signaling were induced by drought, salt and cold^68^. Similar result was obtain in this study, where two *PP2C* genes (PP2C1:Ciclev10028495m and PP2C2: Ciclev10005200m) were strikingly reduced by dehydration and salt stress.

It is well known that transcription factors (TFs) play crucial roles in plant development and stress response^41^. As shown in table 6, at least 8 TFs families were negatively or positively responded to dehydration and salt stress, including WRKY, NAC, CBF, ERF, ZIP, MYB, ZFP and CATMA. Of them, WRKY family has been reported to play an important role in drought and salt stresses, as evidenced by studies in Arabidopsis, rice, soybean and *Thlaspi caerulescens*^69^. Similarly, *NAC* genes were also widely involved in plant tolerance to cold, salt and drought stress^1^,^70^. In addition, there were a growing body of other TFs including CBF, ERF, ZIP, MYB, ZFP and CATMA indentified to have critical roles in plant tolerance to drought and salt stresses^1^,^71^,^72^,^73^. These results indicated that the tolerance of citrus root to salt and dehydration stresses was configured by the integrative functioning of numerous genes operating through a highly coordinated regulatory network.

The different expression of many conserved and newly identified miRNAs in citrus root was induced under dehydration and salt treatments; however major miRNAs were uniquely expressed in a stress treatment (Table 3). It was worthy to note that some DE miRNAs such as cj_MIR160, cj_MIR162, cj_MIR168, cj_MIR398, cj_MIR403 etc. did not lead their targets to significantly different expression (Table 3 and Fig. 6), the reasons of which remain to be elucidated. Despite this, at least 114 DE mRNAs potentially served as DE miRNA targets, which encoded SPLs, NAC, ZIP, laccase and F-box proteins etc. (Table 6).

NF-YA (GmNFYA3) of the NF-Y complex in soybeans was inducible by drought, NaCl and cold, and overexpression of it in *Arabidopsis* leads to enhanced tolerance to drought and elevates sensitivity to high salinity^74^. An *in vivo* experiment in tobacco demonstrated that *GmNFYA3* is the target of miRNA169. Similarly, *NF-YA1* was also predicted as the target of cj_miRNA169l in citrus. Interestingly, *cj_miRNA169l* was significantly down-regulated just by salt but not dehydration, and as expected, *NF-YA1* just positively responded to salt treatment, suggesting *cj_miRNA169l* play a positive role in salt stress but not in dehydration by acting on NF-YA1 in citrus.

miRNA482 have been found to be associated with drought stress, which target genes includes ARA12 and serine-type endopeptidase in cowpea^75^, and α-mannosidase, pectinesterase, sulfate adenylyltransferase, Caspase/cysteine-type endopeptidase, Thaxtomin resistance protein and thaumatin-like protein 1 etc. in cotton^76^. Here, cj_miRAN482 (cj_MIR482a-3p, cj_MIR482b and cj_MIR482c) was significantly up- and/or down-regulated by salt or/and dehydration treatment, and targeted the genes encoding Calcineurin-like phosphoesterase, apoptotic ATPase, DNA-binding storekeeper protein-related transcriptional regulator and NB-ARC domain protein. Among these target genes, just Calcineurin-like phosphoesterase (Ciclev10020590m) and NB-ARC domain protein (Ciclev10024868m) were responded to salt and drought treatment, showing the potential role of miRNA482 in drought and salt stress. Numerous studies showed that miRNA156 was up- or down-regulated by salt, cold and oxidative stresses^77^ and targeted Squamosa promoter-binding protein-like transcription factors (SPL)^78^. As expected, the cj_MIR156b in this study was significantly down-regulated by salt stress, and 4 SPL members as its targets were positively or negatively responded to salt stress, indicating that cj_MIR156 was involved in salt stress through regulating the expression of *SPLs*.

Additionally, a huge number of other dehydration- or salt-responsive genes were identified to be miRNA targets in this study (Table 6), including bZIP, zinc finger protein, calcium-dependent protein kinase 6, AP2/B3-like transcriptional factor, G-box binding factor 3, glutamate receptor and NAC domain containing protein. Most of these target genes may play an important role in drought and salt stress. For example, ZFP1, a cotton CCCH-type zinc finger protein, could interact with GZIRD21A and GZIPR5 to improve salt stress tolerance^79^ and a chrysanthemum Cys2/His2 zinc finger protein gene might serve as an important regulator involved in the salt and drought stress^80^. Here, cj_MIR3946 was predicted to potentially targets salt-responsive zinc finger in citrus. It was reported that a WD40 repeat-containing protein as positive regulator was associated with wheat tolerance to abscisic acid, salt stress and osmotic stress^81^. In this study, three WD40 repeat-like protein genes were targeted by cj_new_MIR 108, cj_new_MIR 197 and cj_MIR399d. All these miRNAs were down-regulated by salt and dehydration stress.

Surely, there were a series of other DE miRNA and its DE targets such as cj_MIR1515/TIR-NBS-LRR, cj_MIR393b/F-box, cj_MIR3946/G-BOX, cj_MIR3951/Leucine-rich repeat protein kinase that might contribute to salt- and drought- tolerance, which all need further studies in future.

## Conclusions

Overall, there were 2574 mRNAs and 76 miRNAs that were differentially expressed in citrus root under salt and/or dehydration treatments. These genes were functionally associated with carbohydrate metabolism, hormone signal transduction, ROS system, and phenylalanine metabolism. Of them, 466 genes could respond not only to salt stress but also to dehydration, showing the molecular basis of dehydration tolerance was, at least in part, common to that of salt tolerance. It was worthy to note that a number of transcript factors genes including NACs, MYBs, CBFs, ERFs, WRKYs, ZFPs, CAMTAs and bZIPs were involved in salt and drought stress, most of them were significantly up-regulated, while a few miRNAs that target these transcript factor genes were down-regulated. Based on abovementioned results, we propose that the citrus roots dealt with the salt and dehydration stress mainly through regulating transcript factors which then integrated carbohydrate metabolism, Polyamines pathway, ROS system and hormone signaling pathway into a complex network. Additionally, we identify a number of miRNAs and genes that might be targets for manipulation. This study enhances our understanding of molecular mechanisms underlying salt- and drought-response of citrus roots.

## Materials and Methods

### Plant materials

The citrus cultivar, *Citrus junos* Siebold cv. ‘Ziyang’, was used in this study. The fruits were collected from the National Citrus Germplasm Repository (NCGR), Citrus Research Institute, Chinese Academy of Agricultural Sciences, Chongqing, China, from which the seeds were fetched. To accelerate seed germination, we removed the seed coat including testa and endopleura. Then, the peeled seeds were placed on culture medium containing nutrients necessary to the growth of citrus seedlings. When the first true leaves were fully developed, uniform seedlings were selected and treated with salt (300 mM) and dehydration. The roots were harvested at 0 h as control, 1 h for dehydration treatment, and 24 h for salt treatment. More than 10 plants were harvested and pooled for each treatment. Plant materials were quick frozen using liquid nitrogen once harvested and kept at −80 °C until RNA extraction.

### RNA preparation and sequencing

Trizol reagent (TransGen, China) was used to extract total RNA from citrus roots according to the manufacturer’s instructions. RNA degradation and contamination were assessed on 1% agarose gel electrophoresis. RNA concentration and integrity were measured with RNA Nano 6000 Assay Kit of the Bioanalyzer 2100 system (Agilent Technologies, CA, USA). RNA purity was checked using the Kaiao Photometer Spectrophotometer K5500 (Kaiao, Beijin, China). For transcriptome library construction, 3 mg of total RNA of each sample was used for the RNA sample preparations. RNA sequencing libraries were prepared for each RNA-seq sample using TruSeq Stranded Total RNA Sample Preparation kit (Illumina, San Diego, USA) and all of the procedures and standards were performed according to the manual supplied with kit. Subsequently, the library preparations were sequenced on an Illumina Hiseq 2500 platform, 100 bp paired-end reads were generated from transcriptome sequencing. For miRNA sequencing, 5 μg of total RNA per sample was used for RNA sample preparations. NEBNext Mulriplex Small RNA library Prep Set for Illumina (NEB, USA) was used for miRNA sequencing library preparation and all of the procedures and standards were performed according to the manual supplied with this kit. After quality control, the library preparations were sequenced on an Illumina Hiseq 2500 platform and 50 bp single-end reads were generated.

### Analyses of RNA-Seq data

Clean reads and count number of three mRNA transcriptome libraries were assessed and summarized using custom Bioperl scripts. With bowtie2 software^82^, all clean reads were mapped back onto clementina genome sequence (Cclementina_182_v1.0) which was downloaded from phytozome database (http://www.phytozome.net). Gene expression analysis is quantified by TopHat program with the option-classic fpkm^83^. The expression level of each gene was represented by the FPKM value which was calculated by the following formula:


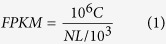


C is the number of fragments that are uniquely aligned to a gene with L bases; N indicates the total number of fragments that are uniquely aligned to all genes.

The P-value between the two samples was calculated using the following formulas^84^:


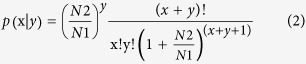


N1 and N2 represent the total clean tag number of the sample 1 and 2, respectively; *x* and y is the tags of a gene in sample 1 and sample 2. The threshold of P-value was adjusted by FDR (False Discovery Rate) method^85^. In this study, genes with FDR ≤ 0.01 and the absolute value of Log_2_^Ratio^ ati were assigned as differentially expressed.

Gene function was annotated according to Nr (NCBI non-redundant protein sequences), Nt (NCBI non-redundant nucleotide sequences), Swiss-Prot (A manually annotated and reviewed protein sequence database), Pfam (Protein family), GO (Gene Ontology), KO (KEGG Ortholog database) and KOG (euKaryotic Ortholog Groups). All the unigenes were searched against Nr, Nt, Swiss-Prot, KO and KOG databases using the BLAST algorithm (E-value < 1E-5). On the basis of GO annotation, the *WEGO* program was used to perform GO functional classification. When a unigene not found in any of the above databases was referred to as novel gene. With a hypergeometric test after Bonferroni Correction (p < 0.05), GO enrichment analysis was performed using a strict algorithm developed based on GO::TermFinder. The method used is described as follow:


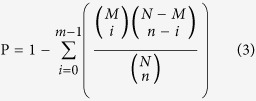


Where N is the number of all genes with GO annotation; n is the number of differentially expressed genes (DEGs) in N; M is the number of all genes that are annotated to certain GO terms; m is the number of DEGs in M. The calculated *p*-value goes through Bonferroni Correction^86^, taking corrected p-value ni Correction (annotation; n is the number of differentially edefined as significantly enriched GO terms in DEGs. KEGG pathway enrichment analysis was done with the same method as that in GO analysis.

### Analysis of miRNA-Seq data

After filtering out the impure sequences (adaptor sequences and the low quality reads) with custom Perl scripts, the cellular structural RNAs, including tRNAs, rRNAs and snoRNAs, were removed using in-house Python scripts. The clean reads were mapped to the clementina genome sequence by Bowtie *et al*.^87^ without mismatch to analyze their expression and distribution on the reference genome. To identify conserved miRNAs, the mapped miRNA tags were then compared with plant mature miRNA sequences which were downloaded from miRBase (http://www.mirbase.org/). Novel miRNA was predicted with software miREAP^88^ and mirdeep2^89^ through exploring the secondary structure, the Dicer cleavage site and the miRNA target prediction minimum free energy of the small RNA tags unannotated in the former steps.

Conserved and novel miRNAs, and clementina genome sequence were used for miRNA target genes prediction by psRobot^90^ and TargetFinder^91^. Differential expression analysis of two samples was performed using DEGseq R package. *P*-value was adjusted using *q*-value^92^. Q-value < 0.01 and log2-fold change ≥ 1 was set as the threshold for significantly differential expression.

### Correlation analysis

To define all the possible miRNA-mRNA interactions, including positive and negative relationships between miRNA and mRNA expression, we use an in-house R script to construct miRNA-mRNA regulatory network. Briefly, normalized all the sample-matched miRNA and mRNA sequencing data; then integration of DE miRNAs with DE mRNAs was achieved by integrating expression profiles of miRNA and mRNA, sample categories and miRNA-targetinginformation to control for false discovery rates.

### qRT-PCR validation of differentially expressed genes and miRNAs

Relative expression levels of the DE genes were quantified by real-time PCR, *actin* gene serving as the internal control. qPCR reactions were performed on an ABI 7300 Fast Real-time PCR System Using iQ SYBR Supremix (Bio-rad, Chengdu, China), 95 °C for 10 min, 40 cycles at 95 °C for 15 s, 60 °C for 15 s, and 72 °C for 15 s. The 10 μL reaction mixture containing 1 μL cDNA, 5 μL 2 × SYBR Green PCR Master Mixture, 0.2 μL each primers (0.1 mM) and 3.6 μL ddH_2_O. With U6 snRNA serving as the internal control, DE miRNAs expression was detected using miRcute Plus miRNA qPCR Detection Kit (TianGen, China). qPCR reactions were performed on an ABI 7300 Fast Real-time PCR System according to the manufacturer’s instructions, 95 °C for 15 min, 40 cycles at 94 °C for 20 s and 60 °C for 34 s. The 20 μL reaction mixture containing 1 μL cDNA, 10 μL 2 × miRcute Plus miRNA Premix (with SYBR & ROX), 0.4 μL each primers, 2 μL 50 × ROX Reference Dye and 6.6 μL ddH_2_O. The 2^−∆∆Ct^ method was employed for relative gene expression level analysis. The primers used for qRT-PCR were listed in Table S2. Triplicates of each reaction were preformed, and student’s t-test was used to analyze the expression difference among samples.

### RNA ligase-mediated 5′ RACE for mapping of mRNA cleavage sites

With Trizol reagent, total RNA was extracted from the *Citrus junos* roots treated by CK, salt and dehydration, respectively and then pooled equally for 5′ RACE. Poly(A)^+^ mRNA was purified using the PolyA kit (Promega, Madison, WI), based on manufacturer’s instructions. RLM-5′ RACE was followed with the GeneRacer Kit (Invitrogen, CA), as described by Song *et al*.^32^. The PCR amplifications were performed using the GeneRacer 5′ primer and the gene-specific primers (Table S4). Nested PCR amplifications were performed using the GeneRacer 5′ nested primer and the nested gene-specific nested primers (Table S4). The amplification products were gel purified, cloned, and sequenced, and at least 6 independent clones were sequenced.

## Additional Information

**How to cite this article**: Xie, R. *et al*. Combined analysis of mRNA and miRNA identifies dehydration and salinity responsive key molecular players in citrus roots. *Sci. Rep.* **7**, 42094; doi: 10.1038/srep42094 (2017).

**Publisher's note:** Springer Nature remains neutral with regard to jurisdictional claims in published maps and institutional affiliations.

## Supplementary Material

Supplementary Dataset 1

Supplementary Dataset 2

## Figures and Tables

**Table 1:** Summary of mRNA sequencing datasets.

Sample	Raw	Clean reads	Error (%)	Paired reads	Mapped reads	Unmapped rate (%)
CK	42,468,660	42,094,647	0.88	41,888,598	35,008,954	16.42
DR	34,424,826	34,102,075	0.94	33,914,741	28,238,492	16.74
SA	37,931,432	37,616,305	0.83	37,436,922	31,414,711	16.09

CK: the control, DR: dehydration, SA: salt.

**Table 2:** Statistics of miRNA sequences of CK, DR and SA cDNA libraries.

	CK library		DR library		SA library	
	Total sRNAs	Unique sRNAs	Total sRNAs	Unique sRNAs	Total sRNAs	Unique sRNAs
Raw reads	18,140,473	—	22,152,310	—	25,460,679	—
High quality reads	18,103,322	—	22,110,220	—	25,397,581	—
Clean reads	16,552,632	2,090,880	19,881,239	2,358,267	23,441,245	1,564,949
Mapping to genome	14,150,794	1,035,128	17,343,294	1,172,809	21,416,519	761,824
Match known miRNAs	2213414	3637	1933427	3622	891277	2898
The unknown sRNAs	72276	**—**	69379	**—**	26939	**—**

CK: the control, DR: dehydration, SA: salt.

**Table 3:** List of 76 DE miRNA in response to dehydration and salt treatments.

Number code	miR_name	log_2_^ratio^	p-value	q-value	Mature sequence	Regulated
1	cj_MIR1515	8.50	3.04E-45	1.90E-44	TCATTTTTGCGTGCAATGATCC	SA
2	cj_MIR156b	−2.46	4.27E-110	4.00E-109	TGACAGAAGAGAGTGAGCAC	SA
3	cj_MIR156e	−1.95	0	0	TTGACGGAAGATAGAGAGCAC	SA
4	cj_MIR156j	−2.39	4.95E-81	3.84E-80	GTGACAGAAGATAGAGAGCGC	SA
5	cj_MIR159	−1.61	0	0	TTTGGATTGAAGGGAGCTCTA	SA
6	cj_MIR160	−1.39	7.24E-30	3.62E-29	GCCTGGCTCCCTGTATGCCAT	SA
7	cj_MIR162	−1.28	4.27E-28	2.09E-27	TCGATAAACCTCTGCATCCAG	SA
8	cj_MIR164	−7.96	8.19E-26	1.48E-25	TGGAGAAGCAGGGCACGTGCA	DR
		−3.20	5.49E-24	2.52E-23		SA
9	cj_MIR164f	−2.78	6.45E-07	1.86E-06	TGGAGAAGCAGGGCACATGCT	SA
10	cj_MIR166c	−1.73	0	0	TCGGACCAGGCTTCATTCCC	SA
11	cj_MIR166d	6.06	3.14E-10	3.14E-10	TCGGACCAGGCTTCATTCCCC	DR
12	cj_MIR166e	−1.97	0	0	TCGGACCAGGCTTCATTCCCC	SA
13	cj_MIR167	−1.53	4.38E-149	4.48E-148	TGAAGCTGCCAGCATGATCTGA	SA
14	cj_MIR168	−1.88	4.96E-108	4.47E-107	TCGCTTGGTGCAGGTCGGGAA	SA
15	cj_MIR169d	6.69	8.96E-15	1.14E-14	GCTAGCCAAGGATGACTTGCCT	DR
16	cj_MIR169i	6.82	5.85E-16	7.68E-16	TAGCCAAGGATGACTTGCCTG	DR
17	cj_MIR169l	−6.26	3.17E-09	1.08E-08	TAGCCAAGGATGACTTGCCTG	SA
18	cj_MIR171	−2.12	8.37E-23	3.77E-22	TTGAGCCGCGTCAATATCTCC	SA
19	cj_MIR171b	−2.06	3.61E-44	2.20E-43	CGAGCCGAATCAATATCACTC	SA
20	cj_MIR2097	5.26	1.97E-06	1.31E-06	TTCTCTTCTTCGAGCGAGAGGT	DR
21	cj_MIR2118	−0.76	9.14E-09	2.98E-08	AATGGGTGCATGGGCAAGAGA	SA
22	cj_MIR319	−2.34	0	0	CTTGGACTGAAGGGAGCTCCT	SA
23	cj_MIR3627	−1.66	3.98E-10	1.42E-09	TTGTCGCAGGAGCGGTGGCACC	SA
24	cj_MIR390	−1.77	3.11E-50	2.06E-49	AAGCTCAGGAGGGATAGCGCC	SA
		5.69	2.94E-08	2.28E-08		DR
25	cj_MIR393	−1.49	3.16E-210	4.18E-209	TTCCAAAGGGATCGCATTGATT	SA
26	cj_MIR393b	−1.39	8.91E-08	2.75E-07	TCCAAAGGGATCGCATTGATC	SA
		0.78	3.00E-05	1.48E-05		DR
27	cj_MIR394	−2.57	8.88E-09	2.94E-08	TTGGCATTCTGTCCACCTCC	SA
28	cj_MIR3946	−6.82	1.03E-12	3.93E-12	TTGTAGAGAAAGAGAAGAGAGCAC	SA
29	cj_MIR3950	−1.91	3.46E-237	4.87E-236	TTTTTCGGCAACATGATTTCT	SA
		−0.8	5.8E-231	5.32E-228		DR
30	cj_MIR3951	−2.03	2.37E-38	1.36E-37	TAGATAAAGATGAGAGAAAAA	SA
		−0.97	1.57E-12	1.86E-12		DR
31	cj_MIR3952	−1.81	0	0	TGAAGGGCCTTTCTAGAGCAC	SA
32	cj_MIR396	−1.79	0	0	TTCCACAGCTTTCTTGAACTG	SA
33		9.47	1.63E-70	4.14E-70		DR
34	cj_MIR397	−2.87	1.43E-14	5.66E-14	TCATTGAGTGCAGCGTTGATG	SA
		−1.52	8.51E-07	5.78E-07		DR
35	cj_MIR398	−2.08	4.20E-293	6.76E-292	AAGGGGTGACCTGAGAACACA	SA
		−1.16	8.16E-115	3.10E-114		DR
36	cj_MIR398b	−2.29	2.00E-22	8.66E-22	GTGTTCTCAGGTCGCCCCTG	SA
		−1.40	3.62E-11	3.93E-11		DR
37	cj_MIR399d	−2.87	1.92E-47	1.24E-46	TGCCAAAGGAGAGTTGCCCTG	SA
		−1.45	3.89E-19	5.49E-19		DR
38	cj_MIR403	−1.29	7.21E-153	7.72E-152	TTAGATTCACGCACAAACTCG	SA
39	cj_MIR408	−2.69	1.47E-34	8.26E-34	ATGCACTGCCTCTTCCCTGGC	SA
		0.72	1.12E-05	6.48E-06		DR
40	cj_MIR472	−2.11	0	0	TTTTTCCCACACCTCCCATCCC	SA
41	cj_MIR473	−2.23	1.75E-106	1.51E-105	ACTCTCCCTCAAGGGCTTCGC	SA
42	cj_MIR477b	−2.88	1.65E-110	1.61E-109	ACTCTCCCTCAAGGGCTTCTCT	SA
43	cj_MIR477c	7.27	1.05E-20	1.54E-20	TCCCTCGAAGGCTTCCAATATA	DR
44	cj_MIR482a-3p	−1.99	0	0	TCTTACCTATGCCACCCATTCC	SA
45	cj_MIR482b	−2.16	7.96E-200	9.42E-199	TCTTGCCCACCCCTCCCATTCC	SA
		1.74	1.50E-194	1.14E-193		DR
46	cj_MIR482c	−1.74	1.33E-164	1.50E-163	TTCCCTAGTCCCCCTATTCCTA	SA
		−11.86	1.02E-207	9.70E-207		DR
47	cj_MIR535	−1.78	2.05E-31	1.07E-30	TGACAATGAGAGAGAGCACAC	SA
		−0.75	2.27E-08	1.80E-08		DR
48	cj_new_MIR016	−1.21	6.96E-33	3.73E-32	GTTGGAGAGCAGCAGTTCGAAC	SA
49	cj_new_MIR027	−6.71	6.26E-12	2.35E-11	TAGCCAAGGATGACTTGCCTGCA	SA
		−6.47	4.27E-11	4.51E-11		DR
50	cj_new_MIR031	−3.95	2.36E-26	1.11E-25	TATGGTACCACAGCTGAATCC	SA
51	cj_new_MIR035	−6.03	4.26E-08	1.35E-07	TTGAGAAGTGTAGTATTATT	SA
52	cj_new_MIR038	−1.86	0	0	TTGCCAACTCCTCCCATGCCGA	SA
53	cj_new_MIR049	−2.38	1.11E-40	6.58E-40	TGAGGCCGTTGGGGAGAGTGG	SA
54	cj_new_MIR052	−2.56	8.12E-11	2.95E-10	TCTGTAACGTAGTTTTGTCCT	SA
55	cj_new_MIR055	−7.84	1.53E-22	6.73E-22	ATCATAGGAAGTAGGCTGCACC	SA
		−7.60	4.43E-21	6.74E-21		DR
56	cj_new_MIR065	−6.51	2.42E-11	2.71E-11	CGACCCGTTAGAACTTTGAAT	DR
		−1.84	8.70E-06	2.15E-05		SA
57	cj_new_MIR091	−6.31	4.24E-10	4.13E-10	AGATCATCTGGCAGTTTCACC	DR
58	cj_new_MIR103	−2.00	8.63E-06	2.16E-05	CTTTCAGCAGCCTCCGGCGTC	SA
59	cj_new_MIR108	−1.94	2.34E-64	1.64E-63	TGTTTTGGGTGAAACGGGTGTT	SA
		−10.28	5.66E-91	1.79E-90		DR
60	cj_new_MIR114	−3.16	4.21E-58	2.87E-57	TTGTCGCCGGAGAGATAGCACC	SA
61	cj_new_MIR119	−5.89	1.61E-07	4.86E-07	ATCGGATCAGGTTGTAAATTC	SA
62	cj_new_MIR125	−2.12	2.57E-201	3.21E-200	AGTTGGTTGGACTCTCGAGAA	SA
63	cj_new_MIR129	−2.16	0	0	TCCCTACTCCACCCATGCCATA	SA
64	cj_new_MIR145	−5.89	1.61E-07	4.86E-07	ATTGAGGATCTTGCTGGAAAC	SA
		−5.66	5.70E-07	4.01E-07		DR
65	cj_new_MIR152	−2.10	5.79E-06	3.55E-06	CTGAAGAGGAATGTTGGTTGT	SA
		5.13				DR
66	cj_new_MIR165	7.02	6.83E-18	9.29E-18	AGGCAGTGATGTTCAGAACTACC	DR
67	cj_new_MIR 166	8.78	2.18E-48	4.89E-48	CCGTAGGTGAACTCTAACATAGC	DR
68	cj_new_MIR 177	5.98	8.47E-10	8.06E-10	TTTCCAGAAATCTTCGTCATC	DR
69	cj_new_MIR 178	6.26	1.67E-11	1.92E-11	ACGTCGTAAACTCGTCTCGTACT	DR
70	cj_new_MIR 197	−2.08	2.95E-11	1.09E-10	TTGAGATTGAAAGTAGTGATT	SA
		−3.45	6.89E-23	1.14E-22		DR
71	cj_new_MIR 198	5.20	3.37E-06	2.17E-06	TGCACGCATGTCAAGATCTGA	DR
72	cj_new_MIR 201	8.30	5.40E-37	1.03E-36	TTCGTGTTCCAATTATTTTTT	DR
73	cj_new_MIR 203	5.74	1.76E-08	1.42E-08	GGATTCGAGTGAAGGACTTGCT	DR
74	cj_new_MIR 219	4.96	8.25E-06	2.09E-05	TCATAGGAAGTAGGCTGCACC	SA
75	cj_new_MIR 227	6.67	7.28E-16	2.92E-15	GGAGGTGCACCCGCCTAAGGTC	SA
76	cj_new_MIR 237	5.54	3.46E-08	1.11E-07	CAAAAGTTAGATTCCTTGGTC	SA

CK: the control, DR: dehydration, SA: salt.

**Table 4:** DE genes related to osmolytes and osmoprotectants.

Genes	Full name	Gene ID	Log2DR/CK	Log2SA/CK	Stresses
**γ-aminobutyric acid**					
GDH2	glutamate dehydrogenase 2	Ciclev10031681m.g	0	2.5	Salt
**Polyamines**					
ADC1	arginine decarboxylase 1	Ciclev10027873m.g	2.0	0	Drought
PAO1	polyamine oxidase 1	Ciclev10016050m.g	0	−2.2	Salt
PAO4	polyamine oxidase 4	Ciclev10011567m.g	2.7	1.3	Drought/Salt
PAO5	polyamine oxidase 5	Ciclev10007864m.g	0	−2.2	Salt
**Starch**, **mono- and disaccharides**					
BMY1	beta-amylase 1	Ciclev10004620m.g	2.4	0	Drought
BMY3	beta-amylase 3	Ciclev10004689m.g	1.2	0	Drought
BMY6	beta-amylase 6	Ciclev10014929m.g	−1.1	0	Drought
**Trehalose**					
TPS11	trehalose hosphatase/synthase 11	Ciclev10007428m.g	1.7	1.9	Drought/Salt
**Raffinose family oligosaccharides**					
GolS1	Galactinol synthase 1	Ciclev10021027m.g	1.6	0	Drought
GolS2	Galactinol synthase 2	Ciclev10001308m.g	6.4	4.5	Drought/Salt
StS1	Stachyose synthase 1	Ciclev10018822m.g	3.8	1.5	Drought/Salt
StS2	Stachyose synthase 2	Ciclev10006437m.g	1.1	0	Drought

CK: the control, DR: dehydration, SA: salt.

**Table 5:** DE genes related to ROS scavenging system and ABA pathway.

Genes	Full name	Gene ID	Log2DR/CK	Log2SA/CK	Stresses
ROS scavenging system					
GST1	glutathione S-transferase zeta 1	Ciclev10002464m.g	−2.0	0	Dehydration
GST7-1	glutathione S-transferase tau 7	Ciclev10005833m.g	1.7	5.3	Dehydration/Salt
GST7-2	glutathione S-transferase tau 7	Ciclev10005835m.g	−2.6	3.0	Dehydration/Salt
GST7-3	glutathione S-transferase tau 7	Ciclev10005850m.g	0	2.6	Salt
GST7-4	glutathione S-transferase tau 7	Ciclev10032686m.g	0	1.3	Salt
GST7-5	glutathione S-transferase tau 7	Ciclev10023959m.g	3.3	0	Dehydration
GST8-1	glutathione S-transferase tau 8	Ciclev10005837m.g	0	2.3	Salt
GST8-2	glutathione S-transferase tau 8	Ciclev10012710m.g	0	2.9	Salt
GST8-3	glutathione S-transferase tau 8	Ciclev10008944m.g	0	2.0	Salt
GST8-4	glutathione S-transferase tau 8	Ciclev10005840m.g	−2.0	0	Dehydration
GST9	glutathione S-transferase tau 9	Ciclev10024585m.g	0	2.8	Salt
GST25	glutathione S-transferase tau 25	Ciclev10002423m.g	−3.4	5.4	Dehydration/Salt
POD1	Peroxidase superfamily protein	Ciclev10017746m.g	−2.5	−6.3	Dehydration/Salt
POD2	Peroxidase superfamily protein	Ciclev10006591m.g	−3.2	−5.9	Dehydration/Salt
POD3	Peroxidase superfamily protein	Ciclev10005432m.g	0	4.4	Dehydration/Salt
POD4	Peroxidase superfamily protein	Ciclev10007121m.g	−2.0	−3.7	Dehydration/Salt
POD5	Peroxidase superfamily protein	Ciclev10032081m.g	0	−3.7	Salt
POD6	Peroxidase superfamily protein	Ciclev10015924m.g	0	−2.3	Salt
POD7	Peroxidase superfamily protein	Ciclev10012179m.g	0	−2.1	Salt
POD8	Peroxidase superfamily protein	Ciclev10012170m.g	0	−2.0	Salt
POD9	Peroxidase superfamily protein	Ciclev10015783m.g	0	−2.0	Salt
POD10	Peroxidase superfamily protein	Ciclev10026035m.g	0	1.4	Salt
Trx1	Thioredoxin superfamily protein	Ciclev10013816m.g	0	−3.0	Salt
Trx2	Thioredoxin superfamily protein	Ciclev10002404m.g	−1.4	0	Dehydration
Trx3	Thioredoxin superfamily protein	Ciclev10017057m.g	2.8	−2.5	Dehydration/Salt
ABA metabolism and signalling					
PP2C1	highly ABA-induced PP2C	Ciclev10028495m.g	3.0	3.7	Dehydration/Salt
PP2C2	highly ABA-induced PP2C	Ciclev10005200m.g	2.2	3.6	Dehydration/Salt
NCED3	9-cis-epoxycarotenoid dioxygenase 3	Ciclev10019364m.g	6.1	4.1	Dehydration/Salt
CYP707A1	cytochrome P450, family 707, subfamily A, polypeptide 1	Ciclev10011655m.g	3.7	0	Dehydration
CYP707A2	cytochrome P450, family 707, subfamily A, polypeptide 2	Ciclev10028346m.g	2.6	1.2	Dehydration/Salt
ABC	ATP-binding cassette 14	Ciclev10011273m.g	−1.3	−2.3	Dehydration/Salt

CK: the control, DR: dehydration, SA: salt.

**Table 6:** Transcription factors differentially expressing under drought and salt stresses.

Genes	Full name	Gene ID	Log2DR/CK	Log2SA/CK	Stresses
WRKY 6	WRKY DNA-binding protein 6	Ciclev10014642m.g	1.5	2.3	Dehydration/Salt
WRKY 11	WRKY DNA-binding protein 11	Ciclev10008836m.g	2	0	Dehydration
WRKY 22	WRKY DNA-binding protein 22	Ciclev10020943m.g	2.5	0	Dehydration
WRKY 23	WRKY DNA-binding protein 23	Ciclev10021174m.g	1.2	0	Dehydration
WRKY 28	WRKY DNA-binding protein 28	Ciclev10018230m.g	0	3.2	Salt
WRKY 33-1	WRKY DNA-binding protein 33	Ciclev10011386m.g	4.7	3.1	Dehydration/Salt
WRKY 33-2	WRKY DNA-binding protein 33	Ciclev10000654m.g	3.0	3.3	Dehydration/Salt
WRKY 35	WRKY DNA-binding protein 35	Ciclev10021624m.g	−1.1	0	Dehydration
WRKY 40-1	WRKY DNA-binding protein 40	Ciclev10008930m.g	5.1	2.5	Dehydration/Salt
WRKY 40-2	WRKY DNA-binding protein 40	Ciclev10009250m.g	0	3.0	Salt
WRKY 40-3	WRKY DNA-binding protein 40	Ciclev10026105m.g	3.3	4.5	Dehydration/Salt
WRKY 41-1	WRKY DNA-binding protein 41	Ciclev10005165m.g	2.4	4.5	Dehydration/Salt
WRKY 41-2	WRKY DNA-binding protein 41	Ciclev10021038m.g	5.3	3.2	Dehydration/Salt
WRKY 43	WRKY DNA-binding protein 43	Ciclev10024257m.g	0	−3.3	Salt
WRKY 46	WRKY DNA-binding protein 46	Ciclev10020744m.g	4.4	0	Dehydration
WRKY 48	WRKY DNA-binding protein 48	Ciclev10005203m.g	2	0	Dehydration
WRKY 50	WRKY DNA-binding protein 50	Ciclev10009761m.g	4.4	2.5	Dehydration/Salt
WRKY 51	WRKY DNA-binding protein 51	Ciclev10026733m.g	3	0	Dehydration
WRKY 70-1	WRKY DNA-binding protein 70	Ciclev10032192m.g	2.5	0	Dehydration
WRKY 70-2	WRKY DNA-binding protein 70	Ciclev10012055m.g	1.1	0	Dehydration
WRKY 74	WRKY DNA-binding protein 74	Ciclev10028715m.g	−1.2	0	Dehydration
WRKY 75	WRKY DNA-binding protein 75	Ciclev10032816m.g	0	2.5	Salt
NAC2-1	NAC domain containing protein 2	Ciclev10001956m.g	3.8	3.8	Dehydration/Salt
NAC2-2	NAC domain containing protein 2	Ciclev10001976m.g	0	1.6	Salt
NAC2-3	NAC domain containing protein 2	Ciclev10019533m.g	2.4	0	Dehydration
NAC9	NAC domain containing protein9	Ciclev10019845m.g	2.2	0	Dehydration
NAC29	NAC domain containing protein 29	Ciclev10032304m.g	3.4	2.7	Dehydration/Salt
NAC31	NAC domain containing protein 31	Ciclev10001403m.g	4.3	0	Dehydration
NAC33	NAC domain containing protein 33	Ciclev10006623m.g	0	−4.1	Salt
NAC036	NAC domain containing protein 36	Ciclev10029007m.g	5.4	2.4	Dehydration/Salt
NAC045	NAC domain containing protein 45	Ciclev10001433m.g	0	−3.3	Salt
NAC047	NAC domain containing protein 47	Ciclev10020717m.g	0	1.4	Salt
NAC058	NAC domain containing protein 58	Ciclev10023578m.g	0	−2.9	Salt
NAC062	NAC domain containing protein 62	Ciclev10019368m.g	3.4	0	Dehydration
NAC071	NAC domain containing protein 71	Ciclev10031966m.g	0	−1.4	Salt
NAC72	NAC domain containing protein 72	Ciclev10008812m.g	4.1	5.3	Dehydration/Salt
NAC84	NAC domain containing protein 84	Ciclev10016434m.g	1.2	0	Dehydration
NAC90	NAC domain containing protein90	Ciclev10029032m.g	3.5	0	Dehydration
CBF4	C-repeat-binding factor 4 (DREB1D)	Ciclev10013766m.g	inf	inf	Dehydration/Salt
CBF2	C-repeat/DRE binding factor 2 (DREB1C)	Ciclev10021923m.g	8.4	0	Dehydration
ERF1-1	ethylene response factor 1	Ciclev10005820m.g	0	3.9	Salt
ERF1-2	ethylene response factor 1	Ciclev10021652m.g	3.2	3.3	Dehydration/Salt
ERF1-3	ethylene response factor 1	Ciclev10021622m.g	0	2.7	Salt
ERF1-4	ethylene response factor 1	Ciclev10016995m.g	0	2.3	Salt
ERF4	ethylene response factor 4	Ciclev10009484m.g	2.9	0	Dehydration
ERF6	ethylene response factor 6	Ciclev10021285m.g	4.0	2.1	Dehydration/Salt
ERF9	ethylene response factor 9	Ciclev10016276m.g	0	1.4	Salt
ERF13-1	ethylene response factor 13	Ciclev10022986m.g	2.8	1.8	Dehydration/Salt
ERF13-2	ethylene response factor 13	Ciclev10024298m.g	3.9	0	Dehydration
ERF48	ethylene response factor 48	Ciclev10032029m.g	2.5	4.3	Dehydration/Salt
HD-ZIP	Homeobox-leucine zipper protein	Ciclev10010326m.g	0	inf	Salt
bZIP5	basic -leucine zipper motif 5	Ciclev10002805m.g	0	1.4	Salt
bZIP17	basic -leucine zipper motif 17	Ciclev10011169m.g	1.4	0	Dehydration
bZIP53	Basic-leucine zipper motif 53	Ciclev10007045m.g	0	1.7	Salt
bZIP58	Basic-leucine zipper motif 58	Ciclev10032777m.g	−1.3	0	Dehydration
bZIP60	basic -leucine zipper motif 60	Ciclev10002005m.g	1.1	0	Dehydration
bZIP61	basic -leucine zipper motif 61	Ciclev10008720m.g	0	−5.3	Salt
bZIPx	Basic-leucine zipper protein	Ciclev10002029m.g	2.4	1.0	Dehydration/Salt
MYB2	myb domain protein 2	Ciclev10021479m.g	inf	inf	Dehydration/Salt
MYB3	myb domain protein 3	Ciclev10009286m.g	0	3.0	Salt
MYB4	myb domain protein 4	Ciclev10028908m.g	−2.1	0	Dehydration
MYB14	myb domain protein 14	Ciclev10021699m.g	0	2.1	Salt
MYB14	myb domain protein 14	Ciclev10017679m.g	1.6	0	Dehydration
MYB15-1	myb domain protein 15	Ciclev10005629m.g	4.0	3.1	Dehydration/Salt
MYB15-2	myb domain protein 15	Ciclev10022057m.g	1.3	0	Dehydration
MYB15-3	myb domain protein 15	Ciclev10022991m.g	1.0	0	Dehydration
MYB36	myb domain protein 36	Ciclev10028804m.g	0	−2.1	Salt
MYB48	myb domain protein 48	Ciclev10029019m.g	−1.1	0	Dehydration
MYB62	myb domain protein 62	Ciclev10015986m.g	0	2.4	Salt
MYB63	myb domain protein 63	Ciclev10005102m.g	−2.1	0	Dehydration
MYB73	myb domain protein 73	Ciclev10029124m.g	1.9	0	Dehydration
MYB77	myb domain protein 77	Ciclev10002239m.g	2.9	0	Dehydration
MYB78	myb domain protein 78	Ciclev10026578m.g	−1.0	0	Dehydration
MYB82	myb domain protein 82	Ciclev10009700m.g	0	−3.1	Salt
MYB85	myb domain protein 85	Ciclev10005666m.g	0	−2.8	Salt
MYB108	myb domain protein 108	Ciclev10005387m.g	0	1.8	Salt
MYB116	myb domain protein 116	Ciclev10021157m.g	0	1.2	Salt
MYB -r1	myb domain protein r1	Ciclev10001979m.g	2.1	0	Dehydration
ZFP1	C2H2-type zinc finger protein	Ciclev10029464m.g	5.1	3.7	Dehydration/Salt
ZFP2	salt tolerance zinc finger	Ciclev10002297m.g	3.8	2.4	Dehydration/Salt
ZFP3	zinc finger (CCCH-type) protein	Ciclev10030920m.g	3.7	0	Dehydration
ZFP4	zinc finger (C3HC4-type RING finger) protein	Ciclev10028738m.g	2.8	0	Dehydration
ZFP5	C2H2-type zinc finger protein	Ciclev10028853m.g	−2.6	0	Dehydration
ZFP6	RING/FYVE/PHD zinc finger protein	Ciclev10021987m.g	2.6	0	Dehydration
ZFP7	RING/FYVE/PHD zinc finger protein	Ciclev10032323m.g	2.4	0	Dehydration
ZFP8	salt tolerance zinc finger	Ciclev10029065m.g	2.2	1.6	Dehydration/Salt
ZFP9	A20/AN1-like zinc finger protein	Ciclev10029439m.g	2.1	0	Dehydration
ZFP10	zinc finger (C5HC2 type) protein	Ciclev10000262m.g	1.8	0	Dehydration
ZFP11	zinc finger protein 4	Ciclev10029351m.g	−1.5	−1.6	Dehydration/Salt
ZFP12	GATA-type zinc finger transcription factor	Ciclev10032018m.g	1.4	0	Dehydration
ZFP13	B-box type zinc finger protein	Ciclev10016798m.g	−1.4	0	Dehydration
ZFP14	BED zinc finger	Ciclev10011114m.g	1.2	0	Dehydration
ZFP15	DOF zinc finger protein 1	Ciclev10026336m.g	1.2	0	Dehydration
ZFP16	zinc finger (CCCH-type) protein	Ciclev10027883m.g	1.1	0	Dehydration
ZFP17	CCCH-type zinc finger protein	Ciclev10014902m.g	1.0	−3.5	Dehydration/Salt
AFP18	Ran BP2/NZF zinc finger-like protein	Ciclev10026703m.g	0	−5.8	Salt
AFP19	C2H2 and C2HC zinc fingers protein	Ciclev10032889m.g	0	3.6	Salt
ZFP20	B-box type zinc finger protein with CCT domain	Ciclev10020440m.g	0	−3.3	Salt
ZFP21	GATA type zinc finger transcription factor	Ciclev10002540m.g	0	−3.1	Salt
ZFP22	C2H2-like zinc finger protein	Ciclev10001255m.g	0	−2.8	Salt
ZFP23	DHHC-type zinc finger protein	Ciclev10019818m.g	0	−2.8	Salt
ZFP24	Zim17-type zinc finger protein	Ciclev10002475m.g	0	−2.5	Salt
ZFP25	zinc finger (C2H2 type) protein	Ciclev10028631m.g	0	−2.3	Salt
ZFP26	mini zinc finger 2	Ciclev10012891m.g	0	−2.1	Salt
ZFP27	salt tolerance zinc finger	Ciclev10029065m.g	0	1.6	Salt
CAMTA1	calmodulin-binding protein	Ciclev10014524m.g	0	3.5	Salt
CAMTA2	Calmodulin binding protein-like	Ciclev10019990m.g	4.4	2.4	Dehydration/Salt
CAMTA3	calmodulin-binding protein	Ciclev10008000m.g	3.0	2.2	Dehydration/Salt
CAMTA4	calmodulin-binding protein	Ciclev10027246m.g	0	−2.2	Salt
CAMTA5	calmodulin-binding protein	Ciclev10000733m.g	4.5	0	Dehydration
CAMTA6	Calmodulin binding protein-like	Ciclev10008603m.g	2.8	0	Dehydration

CK: the control, DR: dehydration, SA: salt.
