# Supplementary material for: Combined analysis of mRNA and miRNA identifies dehydration and salinity responsive key molecular players in citrus roots
Source: Sci Rep. 2017 Feb 6;7:42094. doi: 10.1038/srep42094 (PMC5292693; doi:10.1038/srep42094)
Supplement: Supplementary Dataset 1 [file srep42094-s1.pdf]

Figure S1 The pre-miRNA structue of novel miRNA

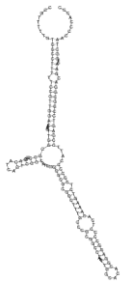

cj_new_MIR001

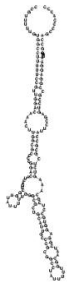

cj_new_MIR002

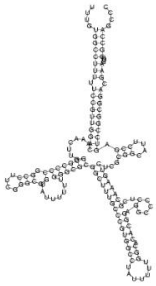

cj_new_MIR003

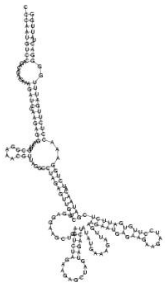

cj_new_MIR004

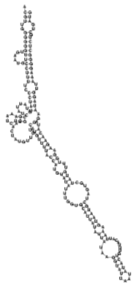

cj_new_MIR005

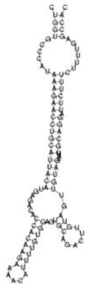

cj_new_MIR006

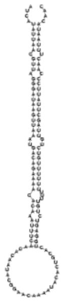

cj_new_MIR007

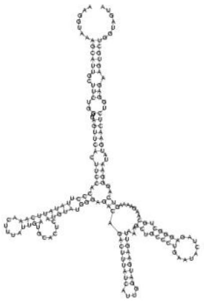

cj_new_MIR008

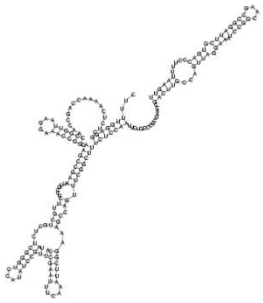

cj_new_MIR009

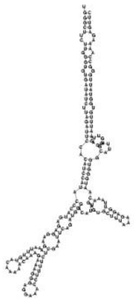

cj_new_MIR010

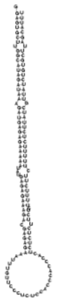

cj_new_MIR011

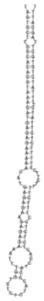

cj_new_MIR012

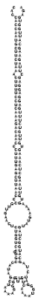

cj_new_MIR013

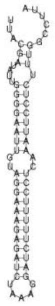

cj_new_MIR014

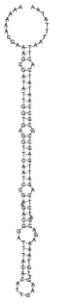

cj_new_MIR015

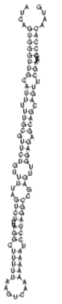

cj_new_MIR016

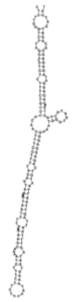

cj_new_MIR017

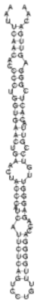

cj_new_MIR018

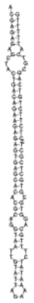

cj_new_MIR019

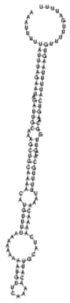

cj_new_MIR020

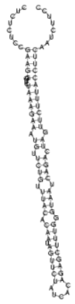

cj_new_MIR021

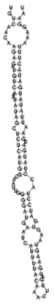

cj_new_MIR022

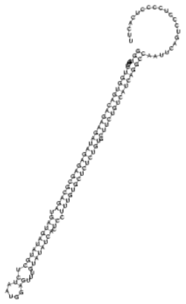

cj_new_MIR023

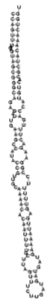

cj_new_MIR024

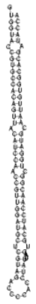

cj_new_MIR025

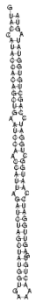

cj_new_MIR026

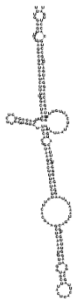

cj_new_MIR027

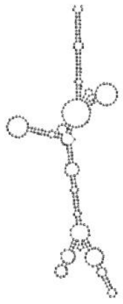

cj_new_MIR028

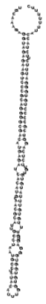

cj_new_MIR029

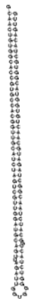

cj_new_MIR030

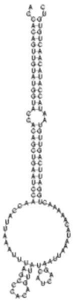

cj_new_MIR031

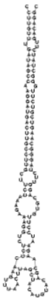

cj_new_MIR032

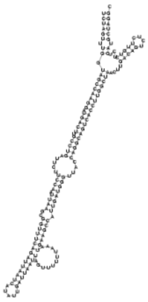

cj_new_MIR033

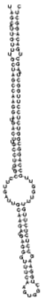

cj_new_MIR034

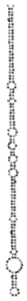

cj_new_MIR035

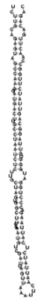

cj_new_MIR036

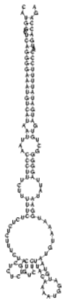

cj_new_MIR037

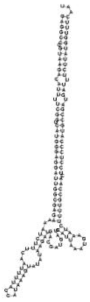

cj_new_MIR038

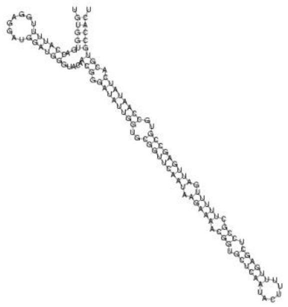

cj_new_MIR039

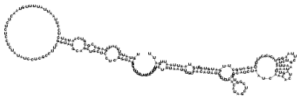

cj_new_MIR040

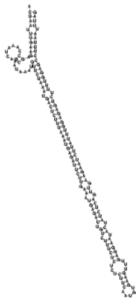

cj_new_MIR041

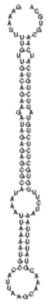

cj_new_MIR042

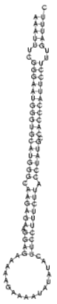

cj_new_MIR043

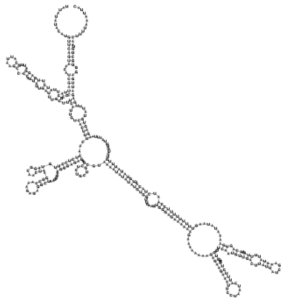

cj_new_MIR044

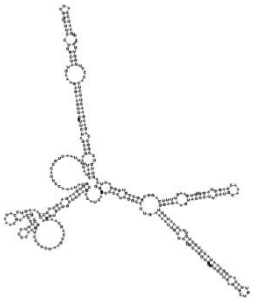

cj_new_MIR045

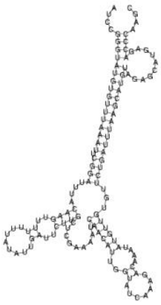

cj_new_MIR046

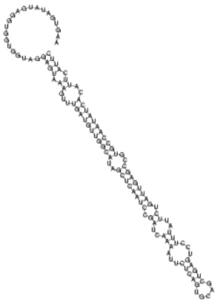

cj_new_MIR047

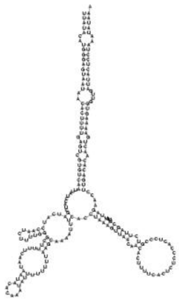

cj_new_MIR048

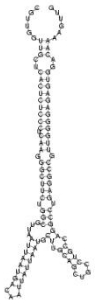

cj_new_MIR049

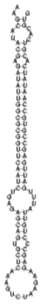

cj_new_MIR050

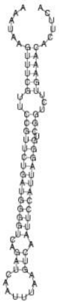

cj_new_MIR051

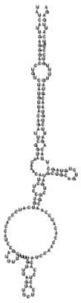

cj_new_MIR052

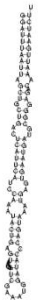

cj_new_MIR053

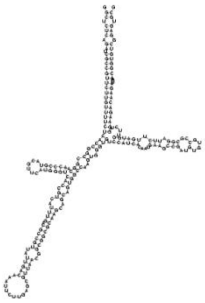

cj_new_MIR054

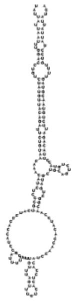

cj_new_MIR055

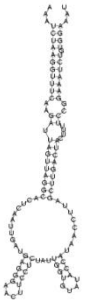

cj_new_MIR056

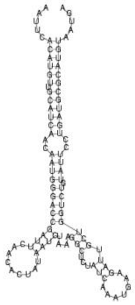

cj_new_MIR057

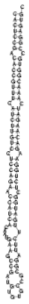

cj_new_MIR058

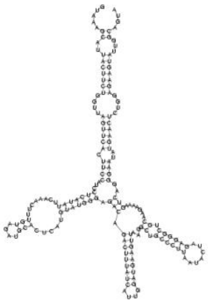

cj_new_MIR059

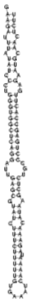

cj_new_MIR060

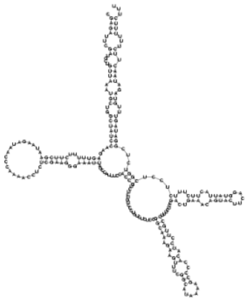
cj_new_MIR061

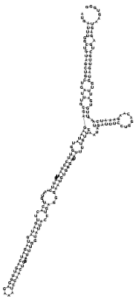
cj_new_MIR062

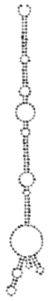
cj_new_MIR063

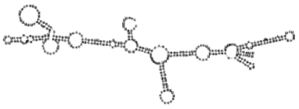
cj_new_MIR064

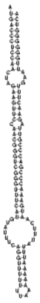
cj_new_MIR065

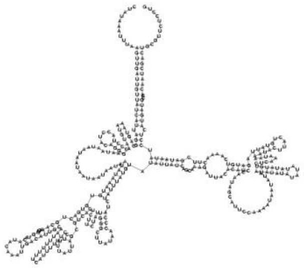
cj_new_MIR066

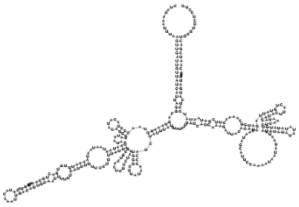
cj_new_MIR067

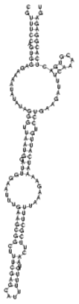
cj_new_MIR068

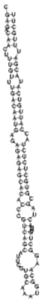
cj_new_MIR069

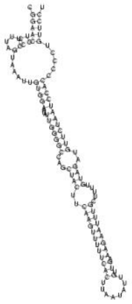
cj_new_MIR070

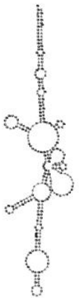
cj_new_MIR071

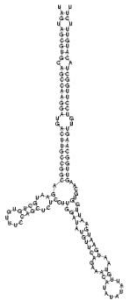
cj_new_MIR072

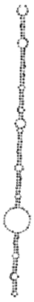
cj_new_MIR073

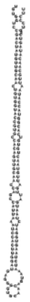
cj_new_MIR074

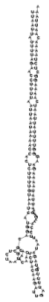
cj_new_MIR075

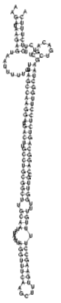

cj_new_MIR076

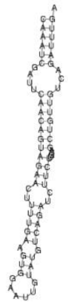

cj_new_MIR077

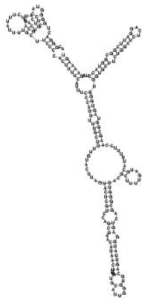

cj_new_MIR078

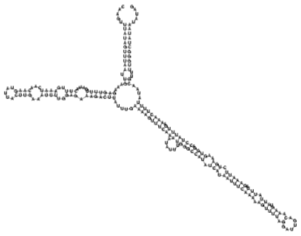

cj_new_MIR079

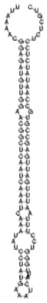

cj_new_MIR080

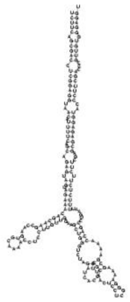

cj_new_MIR081

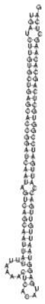

cj_new_MIR082

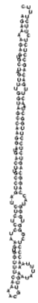

cj_new_MIR083

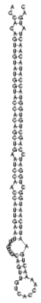

cj_new_MIR084

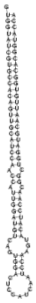

cj_new_MIR085

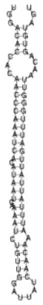

cj_new_MIR086

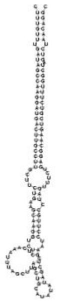

cj_new_MIR087

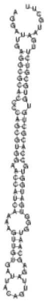

cj_new_MIR088

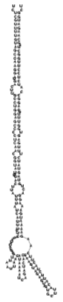

cj_new_MIR089

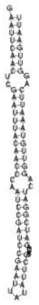

cj_new_MIR090

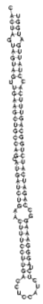
cj_new_MIR091

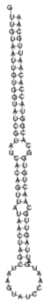
cj_new_MIR092

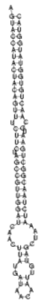
cj_new_MIR093

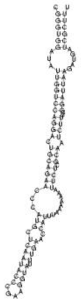
cj_new_MIR094

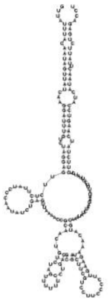
cj_new_MIR095

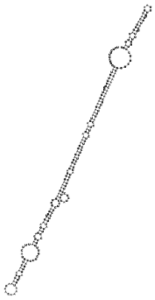
cj_new_MIR096

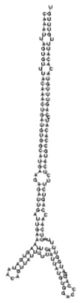
cj_new_MIR097

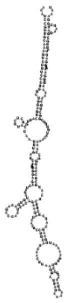
cj_new_MIR098

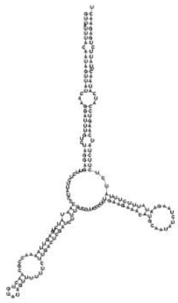
cj_new_MIR099

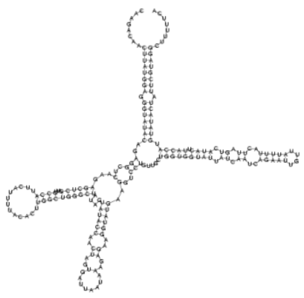
cj_new_MIR100

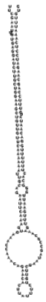
cj_new_MIR101

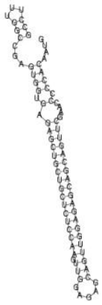
cj_new_MIR102

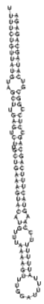
cj_new_MIR103

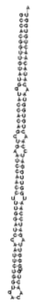
cj_new_MIR104

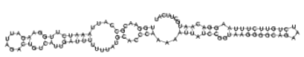
cj_new_MIR105

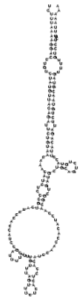

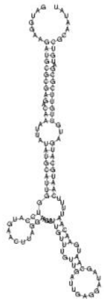

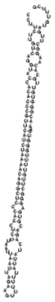

cj_new_MIR106

cj_new_MIR107

cj_new_MIR108

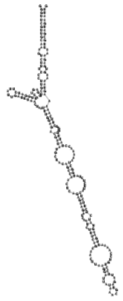

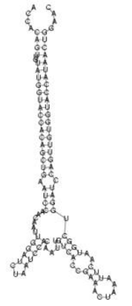

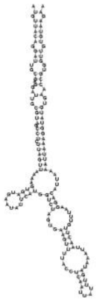

cj_new_MIR109

cj_new_MIR110

cj_new_MIR111

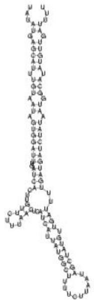

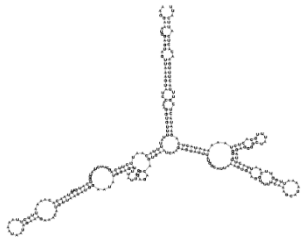

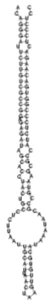

cj_new_MIR112

cj_new_MIR113

cj_new_MIR114

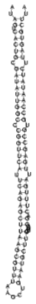

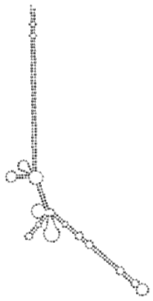

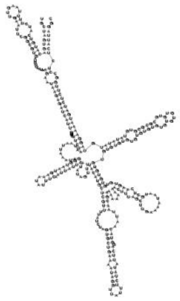

cj_new_MIR115

cj_new_MIR116

cj_new_MIR117

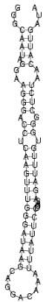

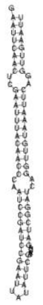

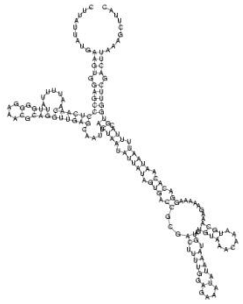

cj_new_MIR118

cj_new_MIR119

cj_new_MIR120

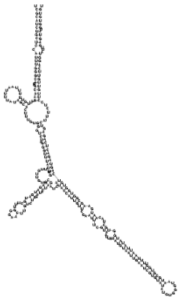

cj_new_MIR121

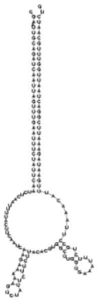

cj_new_MIR122

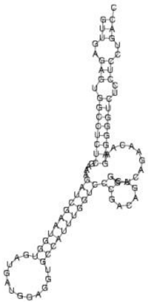

cj_new_MIR123

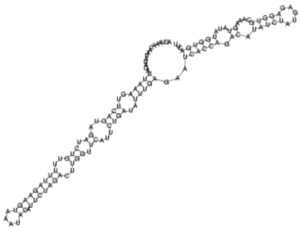

cj_new_MIR124

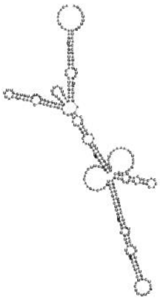

cj_new_MIR125

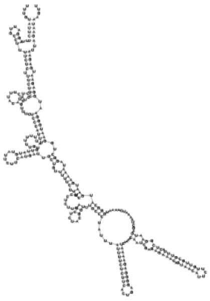

cj_new_MIR126

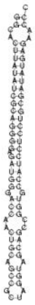

cj_new_MIR127

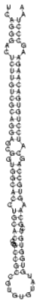

cj_new_MIR128

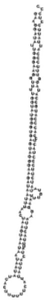

cj_new_MIR129

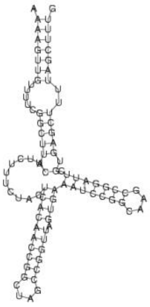

cj_new_MIR130

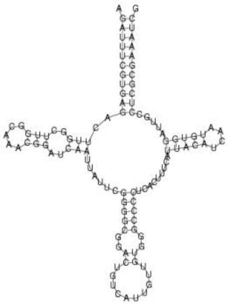

cj_new_MIR131

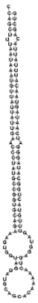

cj_new_MIR132

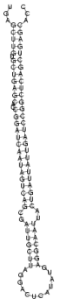

cj_new_MIR133

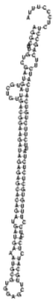

cj_new_MIR134

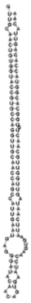

cj_new_MIR135

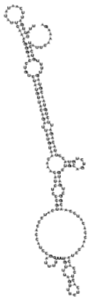
cj_new_MIR136

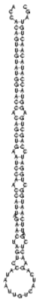
cj_new_MIR137

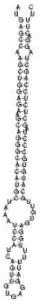
cj_new_MIR138

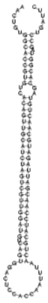
cj_new_MIR139

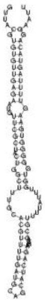
cj_new_MIR140

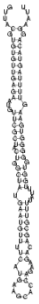
cj_new_MIR141

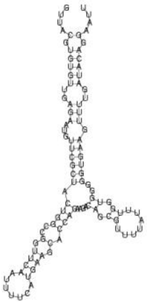
cj_new_MIR142

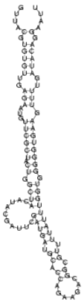
cj_new_MIR143

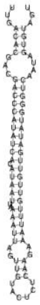
cj_new_MIR144

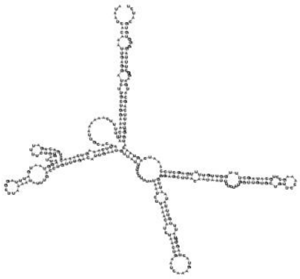
cj_new_MIR145

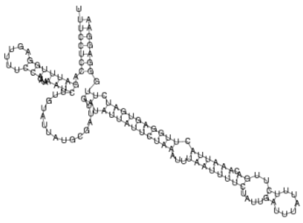
cj_new_MIR146

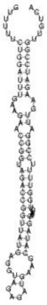
cj_new_MIR147

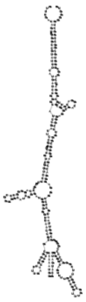
cj_new_MIR148

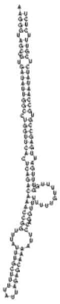
cj_new_MIR149

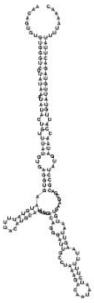
cj_new_MIR150

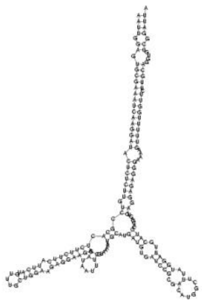

cj_new_MIR151

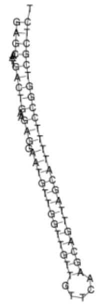

cj_new_MIR152

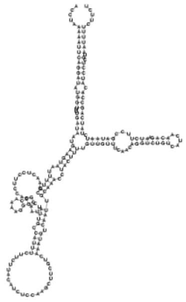

cj_new_MIR153

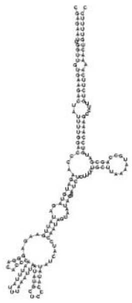

cj_new_MIR154

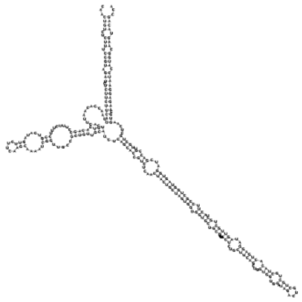

cj_new_MIR155

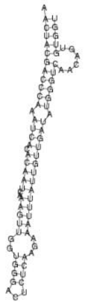

cj_new_MIR156

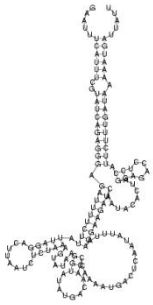

cj_new_MIR157

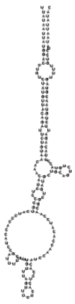

cj_new_MIR158

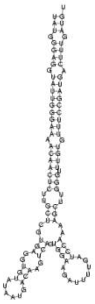

cj_new_MIR159

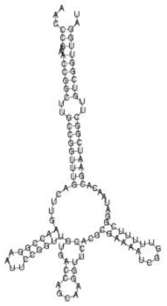

cj_new_MIR160

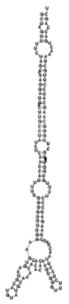

cj_new_MIR161

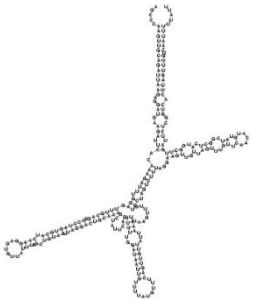

cj_new_MIR162

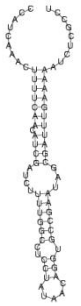

cj_new_MIR163

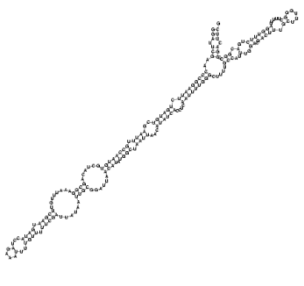

cj_new_MIR164

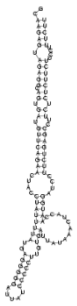

cj_new_MIR165

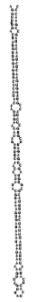
cj_new_MIR166

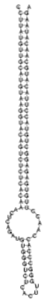
cj_new_MIR167

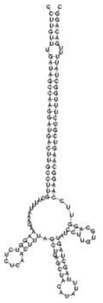
cj_new_MIR168

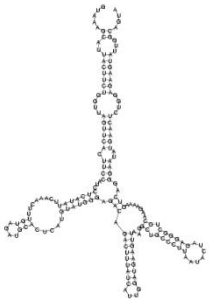
cj_new_MIR169

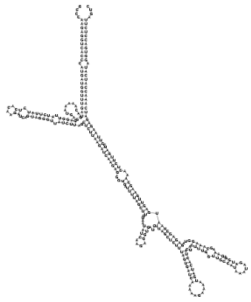
cj_new_MIR170

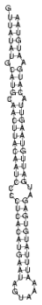
cj_new_MIR171

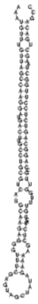
cj_new_MIR172

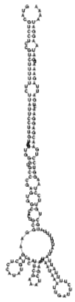
cj_new_MIR173

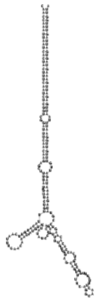
cj_new_MIR174

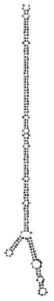
cj_new_MIR175

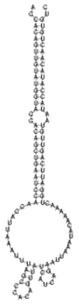
cj_new_MIR176

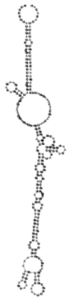
cj_new_MIR177

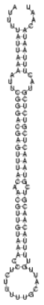
cj_new_MIR178

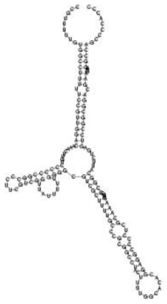
cj_new_MIR179

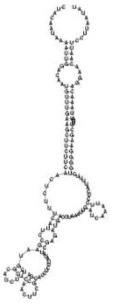
cj_new_MIR180

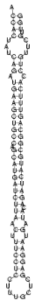

cj_new_MIR181

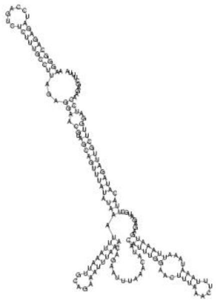

cj_new_MIR182

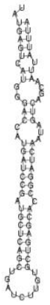

cj_new_MIR183

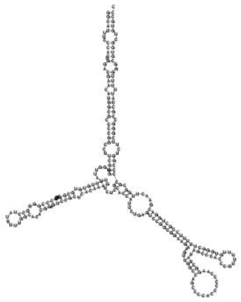

cj_new_MIR184

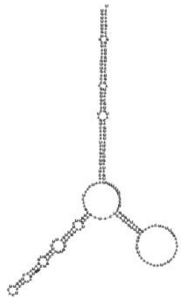

cj_new_MIR185

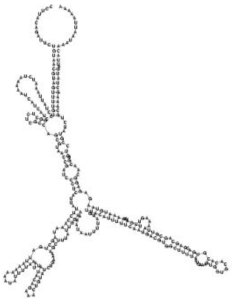

cj_new_MIR186

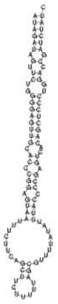

cj_new_MIR187

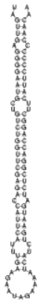

cj_new_MIR188

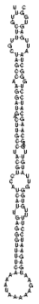

cj_new_MIR189

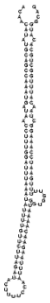

cj_new_MIR190

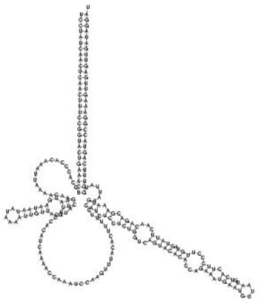

cj_new_MIR191

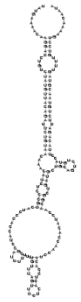

cj_new_MIR192

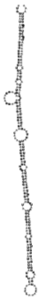

cj_new_MIR193

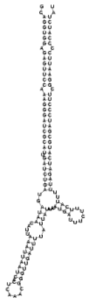

cj_new_MIR194

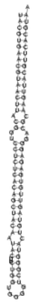

cj_new_MIR195

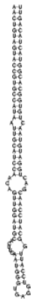

cj_new_MIR196

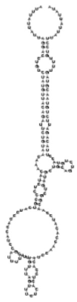

cj_new_MIR197

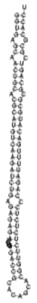

cj_new_MIR198

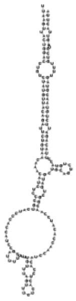

cj_new_MIR199

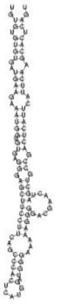

cj_new_MIR200

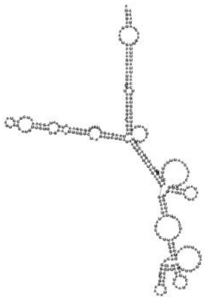

cj_new_MIR201

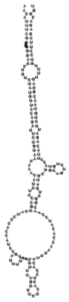

cj_new_MIR202

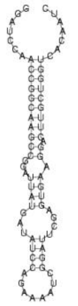

cj_new_MIR203

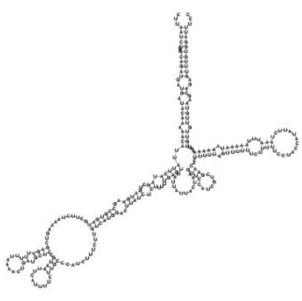

cj_new_MIR204

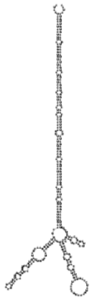

cj_new_MIR205

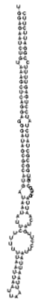

cj_new_MIR206

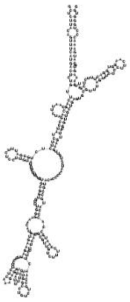

cj_new_MIR207

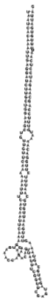

cj_new_MIR208

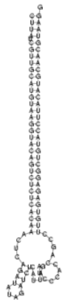

cj_new_MIR209

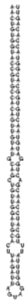

cj_new_MIR210

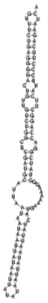

cj_new_MIR211

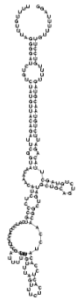

cj_new_MIR212

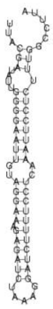

cj_new_MIR213

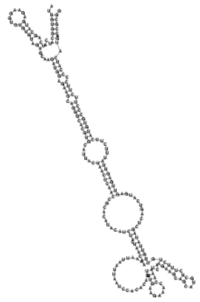

cj_new_MIR214

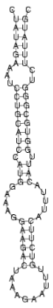

cj_new_MIR215

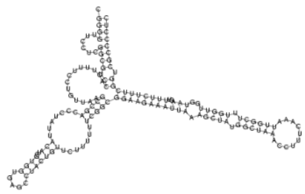

cj_new_MIR216

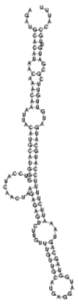

cj_new_MIR217

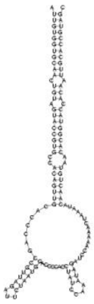

cj_new_MIR218

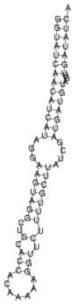

cj_new_MIR219

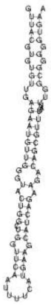

cj_new_MIR220

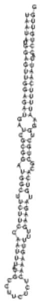

cj_new_MIR221

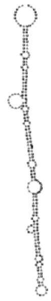

cj_new_MIR222

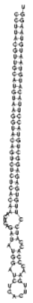

cj_new_MIR223

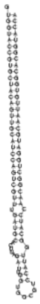

cj_new_MIR224

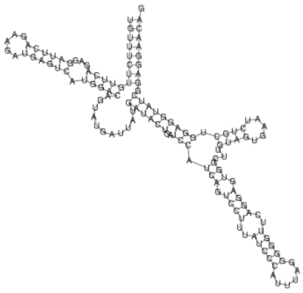

cj_new_MIR225

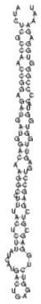

cj_new_MIR226

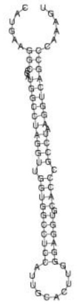

cj_new_MIR227

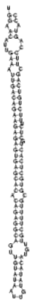

cj_new_MIR228

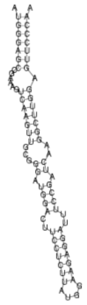

cj_new_MIR229

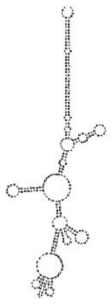

cj_new_MIR230

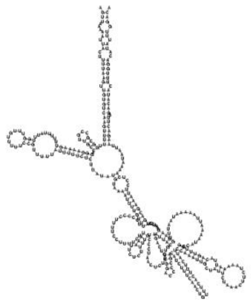

cj_new_MIR231

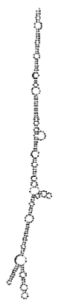

cj_new_MIR232

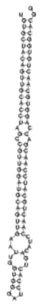

cj_new_MIR233

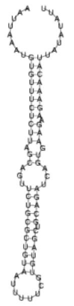

cj_new_MIR234

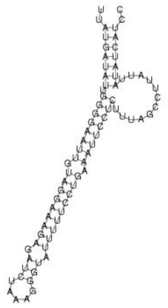

cj_new_MIR235

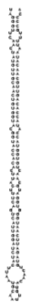

cj_new_MIR236

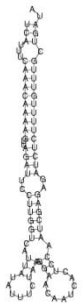

cj_new_MIR237

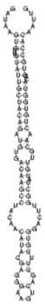

cj_new_MIR238

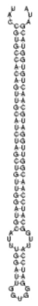

cj_new_MIR239

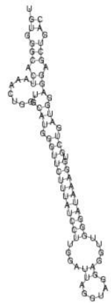

cj_new_MIR240

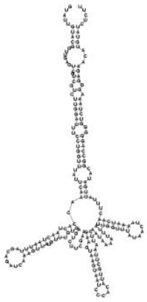

cj_new_MIR241

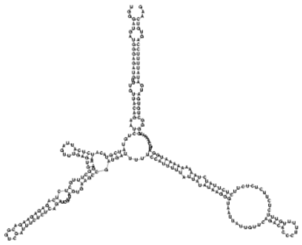

cj_new_MIR242
